# Fluorinated azobenzenes as supramolecular halogen-bonding building blocks

**DOI:** 10.3762/bjoc.15.197

**Published:** 2019-08-23

**Authors:** Esther Nieland, Oliver Weingart, Bernd M Schmidt

**Affiliations:** 1 Institut für Organische Chemie und Makromolekulare Chemie, Heinrich-Heine-Universität Düsseldorf, Universitätsstraße 1, D-40225 Düsseldorf, Germany; 2 Institut für Theoretische Chemie und Computerchemie, Heinrich-Heine-Universität Düsseldorf, Universitätsstraße 1, D-40225 Düsseldorf, Germany

**Keywords:** azobenzene, DFT calculations, fluorine chemistry, halogen bonding, photochemistry

## Abstract

*ortho*-Fluoroazobenzenes are a remarkable example of bistable photoswitches, addressable by visible light. Symmetrical, highly fluorinated azobenzenes bearing an iodine substituent in *para*-position were shown to be suitable supramolecular building blocks both in solution and in the solid state in combination with neutral halogen bonding acceptors, such as lutidines. Therefore, we investigate the photochemistry of a series of azobenzene photoswitches. Upon introduction of iodoethynyl groups, the halogen bonding donor properties are significantly strengthened in solution. However, the bathochromic shift of the π→π* band leads to a partial overlap with the n→π* band, making it slightly more difficult to address. The introduction of iodine substituents is furthermore accompanied with a diminishing thermal half-life. A series of three azobenzenes with different halogen bonding donor properties are discussed in relation to their changing photophysical properties, rationalized by DFT calculations.

## Introduction

The halogen bond is an attractive noncovalent interaction between a polarized halogen atom (the halogen bond donor) and a Lewis base (the halogen bond acceptor) [1–2]. A prominent example regarding the origin of halogen bonding can be found in inorganic solid-state chemistry. The structurally diverse group of polyiodides, with its rich structural chemistry is governed by halogen bonding, where I^−^ and I_3_^−^ are considered the nucleophilic (halogen bond acceptor) and I_2_ the electrophilic (halogen bond donor) subcomponent [3–7]. Neutral halogen bonds on the other hand can be generally described by R–X···Y, where R–X is the halogen bond donor, R is covalently bound to X, and Y is the Lewis basic halogen bond acceptor [1]. In recent years, halogen bonding was used to assemble molecules, leading to a variety of supramolecular architectures [8–19], as well as discrete supermolecules [20–24]. Huber and co-workers demonstrated the activation of a carbonyl group by halogen bonding, and successfully applied this concept to catalysts for Michael addition reactions [25] and also employed neutral [26], and hypervalent iodolium derivatives as activators in a halide abstraction reaction and as organocatalysts in Diels–Alder reactions [27]. The group of Metrangolo also reported halogen bonding-promoted catalysis in water by exploiting a halogen bonding amino acid, which combines excellent donor properties with good water solubility [28], in addition to their seminal contributions in the field of crystal engineering [1]. Only few supramolecular capsules were reported so far [29], including the resorcin[4]arene capsules of Diederich and co-workers [21,23], triangular macrocycles assembled by self-complemented halogen bonding [20] and halogen bond templated, polyfluorinated stilbene squares used for topochemical polymerization [22]. Additionally, halonium ions [N···I^+^···N] were reported to form several charged, discrete supramolecular capsules [30–33] and helicates [34]. In the same line, we have demonstrated recently that both *E*-4,4’-di(iodo)perfluoroazobenzene (**A2**) and *E*-4,4’-di(iodoethynyl)perfluoroazobenzene (**A3**) halogen bond donors can be combined with rigid u-shaped anthracene building blocks, bearing two 3,5-lutidine acceptors in 1 and 8 positions, to form self-assembled boxes of 25–30 Å length in solution and in the solid state [35].

We chose azobenzene because azobenzene is one of the simplest molecules that can undergo photoinduced isomerisation of its N=N central double bond. The photoisomerisation reaction in n→π and π →π* excited states has been studied with experimental [36–38] and theoretical approaches [39–44]. By substituting azobenzenes in the *ortho*-positions to the N=N bond with electron-withdrawing fluorine substituents [45–46], the red-shifting of the n→π* transitions enables selective addressing of both the *E*- and *Z*-isomer using visible light. Stabilization of the n-orbitals in the *Z*-isomers leads to a very high thermal stability of the *Z*-isomer, now exhibiting thermal half-lives up to two years at room temperature [36]. Most important for application in supramolecular systems, it should be possible to study both states of the system on a laboratory timescale, a key aspect in the design of our halogen bonded boxes [35]. Therefore, we herein present a comprehensive investigation of the photochemistry of highly fluorinated azobenzenes. Our efforts are supported by theoretical calculations, showing that these azobenzenes are suitable for the use as building blocks in supramolecular architectures.

## Results and Discussion

**Scheme 1:**
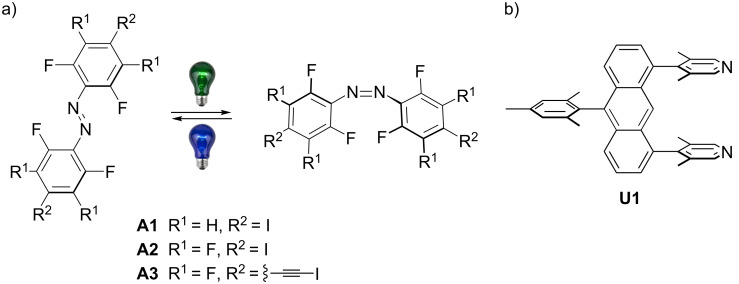
a) Azobenzenes **A1**–**3** employed in this study. b) U-shaped anthracene halogen bond acceptor bearing two 3,5-lutidines in 1 and 8 positions.

Three different azobenzenes **A1**–**3** were studied with regard to halogen bonding and photochemical properties (Scheme 1).

**Figure 1:**
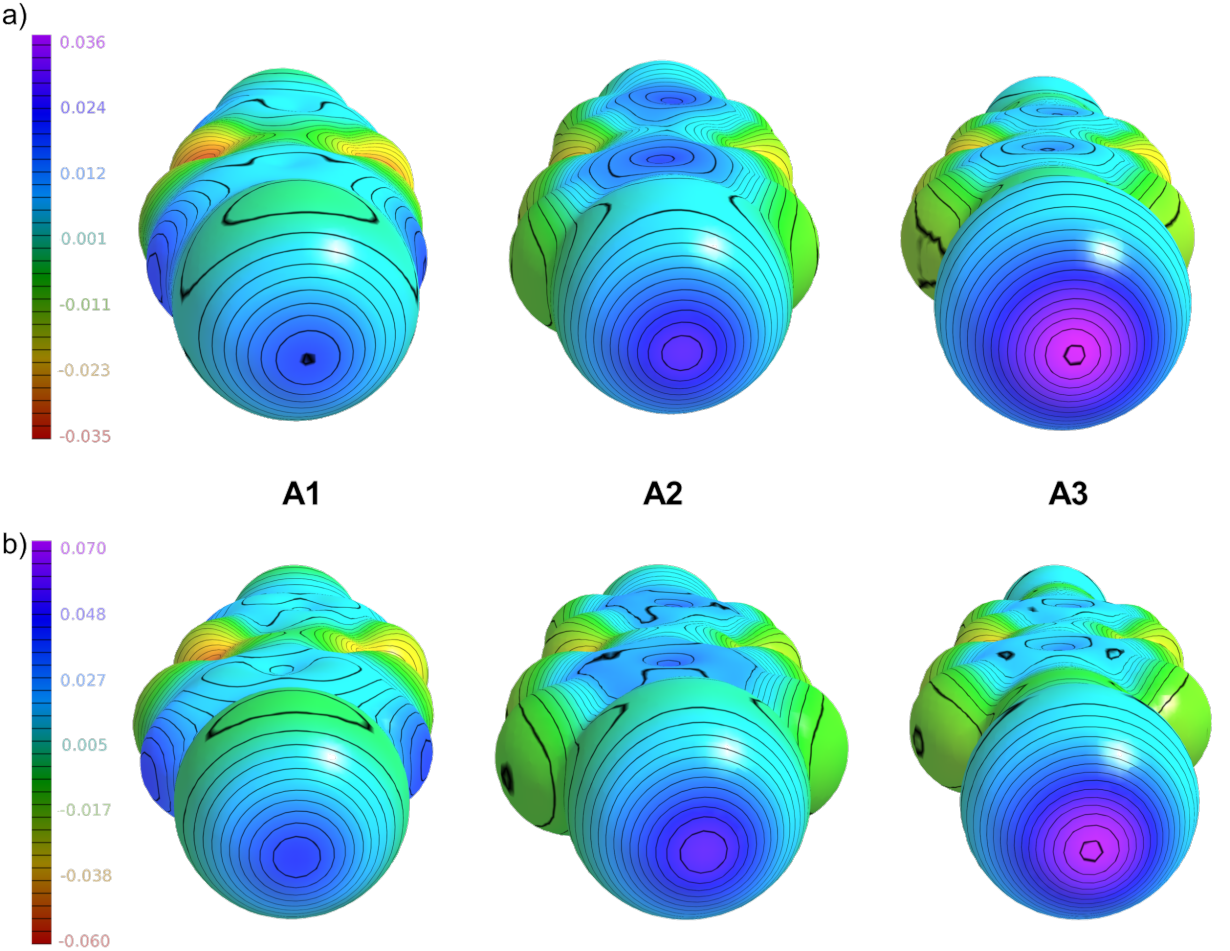
Electrostatic potential map at different isodensity values (B3LYP/ def2/TZVP/DGZVP optimized geometries) with a) *ρ* = 0.0001, and b) *ρ* = 0.001. For visualization, the MoleCoolQt software was used [47].

In our experiments, tetrafluorinated **A1** does not form halogen bonded boxes with acceptor **U1**, neither in solution, nor in the solid state. Octafluorinated **A2** forms [2 + 2] boxes in the solid state and possibly in solution, whereas tetrafluoroiodoethynyl **A3** is as donor strong enough to reliably permit the characterization of the boxes formed in solution and in the solid state [35]. Electrostatic interaction plays a dominant role in halogen bonding [1,12–13]. Therefore, we calculated the molecular electrostatic potentials of the halogen bond donors **A1**–**3** to visualize their capabilities to form halogen bonded architectures (Figure 1).

**Figure 2:**
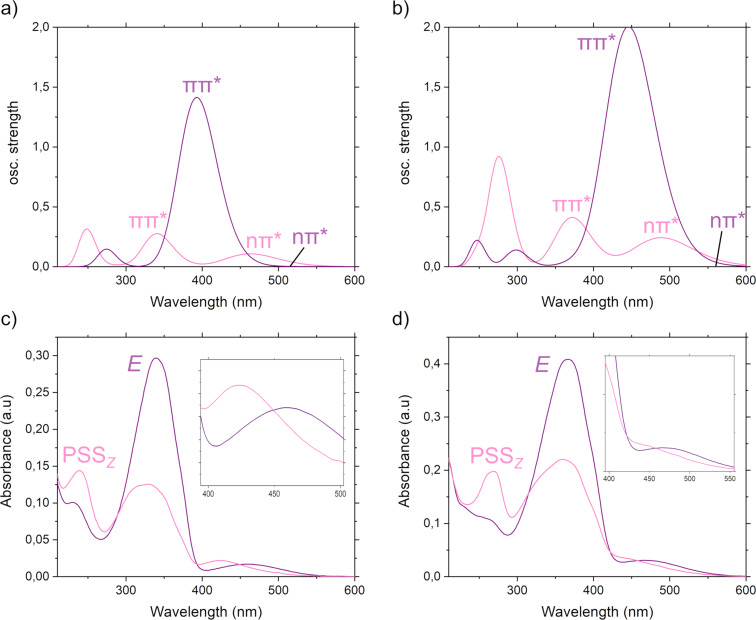
Top: Vertical electronic absorption spectra of a) **A2** and b) **A3**, calculated using TD-B3LYP/def2-TZVP level of theory with Grimme D3 dispersion corrections and implicit MeCN solvent. Pink line: *Z*-isomer, purple line: *E*-isomer. Bottom: *E*-state of c) **A2** and d) **A3** (from left to right, purple) and photostationary state (PSS, pink) after photoirradiation with λ_irr_ = 565 nm, monitored by UV–vis (MeCN, *c* = 10.5 and 9.3 μmol/L, respectively). The inlets of c) and d) were smoothed using the Savitzky–Golay filter implemented in OriginPro to facilitate readability.

Looking at the electrostatic surface potentials of the halogen bond donors, one can see that **A3** shows a maximum value on the iodine atom that is most positive compared to that of **A2** and **A1**. The evolution of the iodine potential follows our experimental observation with iodoethynylazobenzene **A3** being the strongest halogen bond donor and **A1** being the weakest, within this series [48]. For potential, reversible photochemical control of supramolecular assemblies, the halogen donors need to bind both in the *E*- and *Z*-state. This is the case according to our computations, as isodensity values remain almost identical upon switching (Figure S14 in Supporting Information File 1). We therefore turned our attention to elucidate the change of photochemical properties upon introducing the heavy iodine to the azobenzene building block, as well as the effect of the ethynyl group in case of **A3** (Figure 2). Vertical electronic absorption spectra of the different azobenzenes were calculated at the TD-B3LYP/def2-TZVP level of theory including Grimme D3 dispersion correction, using the Gaussian 16 program package (see Supporting Information File 1). The azobenzenes were embedded in a continuum using the polarizable continuum model (PCM) for the solvent MeCN. The DGZVP all electron basis was used for iodine. Vertical excitation energies for the π→π* and n→π* transitions of *E* and *Z*-isomers are listed in the Table S9 (Supporting Information File 1).

The computational absorption spectra are in fair agreement with the experimental ones (Table 1) and trends are reproduced accordingly (measured and calculated absorption spectra of **A1** can be found in the Supporting Information File 1, Figures S1 and S12). By introduction of fluorine atoms *ortho* to the azo bond, the two n→π* of *E*- and *Z*-state become sufficiently separated to address them individually using visible light sources. Along with averting UV light for the photochemical reaction, high PSS ratios can be observed, which is very desirable for application in supramolecular systems [12–13,35]. Tetra- and octafluorinated **A1** and **A2** show clear spectral separation of the n→π* bands, whereas the extended π-system of iodoethynyl **A3** lead to a bathochromic shift of the π→π* band by 24 nm, now partially overlapping with the, also broadened, n→π* band of *Z*-**A3**. Apart from the photoisomerisation using light, azobenzenes **A1**–**3** also undergo thermal back reaction, which we studied experimentally and theoretically. To gain insight into the effect of the iodine atoms on the thermal stability we investigated **A1**, **A2** and **A3** in MeCN at elevated temperature (60 °C), following the works of Hecht and co-workers [45–46]. The data is presented in Table 2. Additionally, we studied **A3** in a wide range of temperatures in MeCN.

**Table 1:** Spectroscopic properties in MeCN for *E-*state and PSS after photoirradiation with λ_irr_ = 565 nm. Maxima were determined using the “Peak Analyzer” implemented in OriginPro. Kinetic measurements were performed in MeCN at 60 °C (Supporting Information File 1).

	λ_max_(*E*) [nm]	ε_max_(*E*) [M^−1^ cm^−1^]	λ_max_(*PSS_Z_*) [nm]	ε_max_(*PSS_Z_*) [M^−1^ cm^−1^]	τ_1/2_ [h]

**A1**	335	3.48 × 10^4^	241	1.68 × 10^4^	44.92
**A2**	340	2.84 × 10^4^	239	1.38 × 10^4^	17.17
**A3**	364	4.41 × 10^4^	359	2.38 × 10^4^	0.92

**Table 2:** Activation process parameters for the *E*→*Z* isomerisation in MeCN at 60 °C, B3LYP, def-TZVP basis for C, H, N, F, DGZVP all electron basis for iodine. Grimme D3 dispersion correction was applied. Values were computed using the KistHelp program [49] employing classical transition state theory and including the effects of Wigner-tunnelling (Supporting Information File 1).

	Δ*U* [kJ mol^−1^]	Δ*G* [kJ mol^−1^]	Δ*H* [kJ mol^−1^]	Δ*S* [J mol^−1^]	*k*_Z-_*_E_* [s^−1^]	τ_1/2_ [h]	τ_1/2_ exp. [h]

**A1**	124.10	114.32	119.06	14.22	9.46 × 10^−6^	20.35	44.92
**A2**	118.86	108.73	114.49	17.28	7.06 × 10^−6^	2.73	17.17
**A3**	113.13	99.89	108.69	26.42	1.70 × 10^−6^	0.11	0.92

The half-lives decrease from **A1** to **A3**, an effect that correlates with the increase in dipole moment of the transition state (TS, see Supporting Information File 1, Table S8). In the B3LYP computations this value is larger than for the corresponding *Z*-isomer and leads to a stabilization of the TS in polar solvents [46].

The bistable character is obviously weakened upon improving the halogen bonding properties. However, most importantly, the azobenzenes still can be conveniently handled at room temperature with a half-life of at least a working day, allowing for studying both states of the systems without needs for in situ irradiation (the thermal half-life of **A3** at room temperature is 14.98 hours in MeCN, see the Supporting Information File 1, Figures S6–S8).

**Figure 3:**
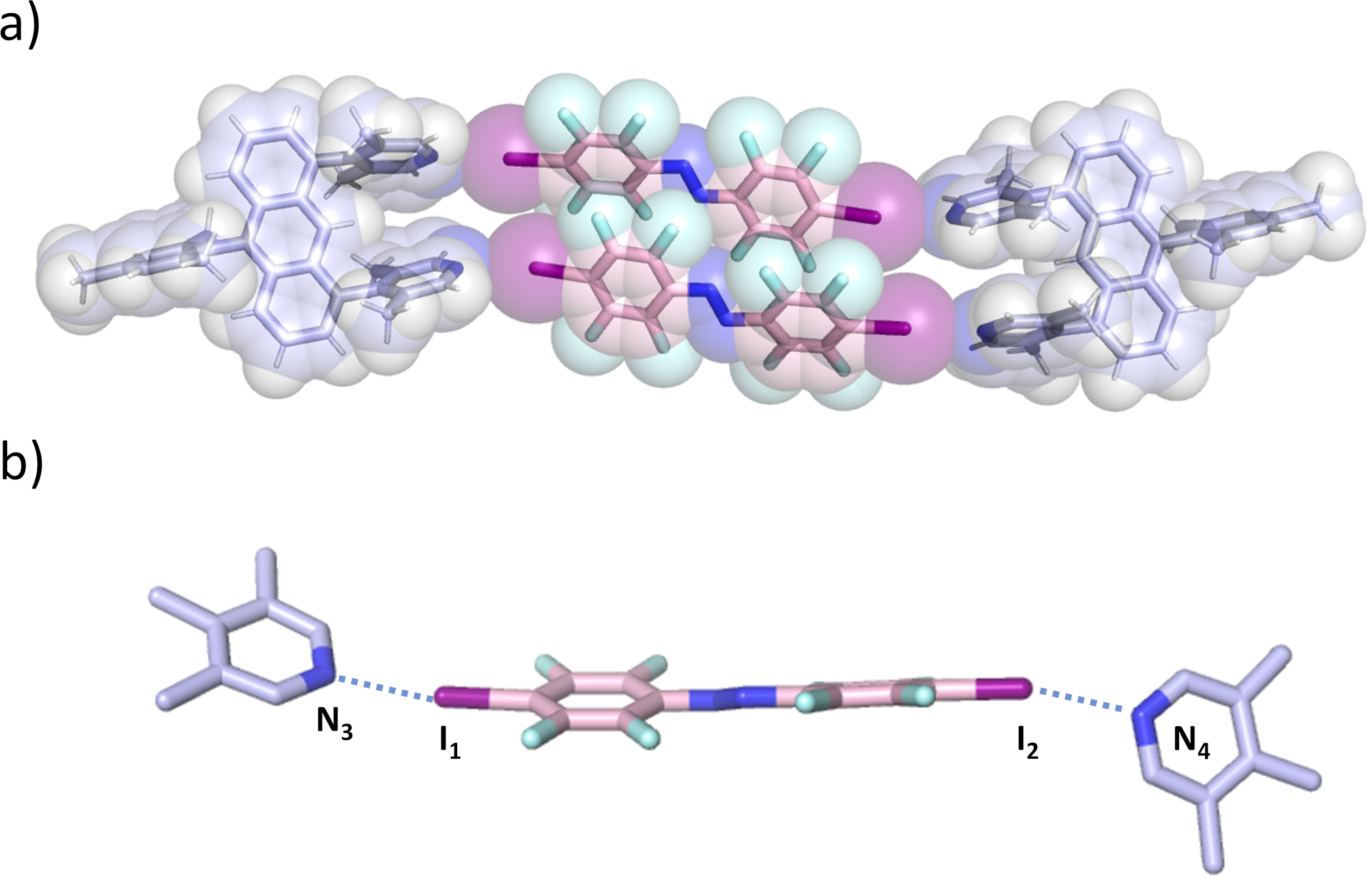
a) Space-filling model of **U1**···**A2**. The kinked alignment of both the lutidine units of **U1** and the azobenzenes **A2** can be seen. b) Part of the X-ray crystal structure showing the halogen bonding azobenzene **A2** in detail. Selected bond lengths: N_3_–I_1_ 2.7810(2), I_2_–N_4_ 2.816(2) Å.

In addition to that, slow evaporation of an equimolar solution of **U1** and **A2** in benzene furnished red-orange single crystals suitable for X-ray analysis of a [2 + 2] halogen-bonded box, **U1**···**A2**, over the course of a few days in quantitative yield. Single-crystal analysis confirms the formation of a **U1**···**A2** box in the solid state (Figure 3).

The **U1**···**A2** box has a principal length of approximately 25 Å (anthracene–anthrance distance) and a height of 5 Å (distance between the *ipso*-carbons of the lutidines). The lutidine acceptor units are curved inwards (with N···I–C angles of 165 and 172°) and show N···I distances of 2.78 and 2.82 Å to the azobenzene donors. As observed for the other boxes assembled by halogen bonding reported by us [35], parts containing fluorinated azobenzenes **A2** are segregated from the perhydrogenated anthracene **U1** units, connected by C–H···F contacts. The azobenzenes **A2** interact by lamellar 2D π-stacking, anthracene **U1** interact predominantly by C–H···π interactions as both the solubilizing mesitylene group and the two perpendicular lutidine acceptors effectively prevent stacking of the anthracenes body (Supporting Information File 1, Figure S17). This was also the key to being able to lower the temperature to characterize formation in the ^1^H NMR, where the solubility of the assemblies in benzene was improved by adding a solubilizing mesitylene group to the halogen bonding acceptor **U1** to avoid precipitation of box **A2**···**U1** during previous titration experiments [35].

## Conclusion

The performed calculations show that both *E-* and *Z*-isomer are equally able to undergo halogen bonding. By improving the strength of halogen bonding going from tetrafluorinated **A1** to octafluorinated **A2** to **A3**, especially by introducing the iodoethynyl group, as trade-off, photophysical properties are changing. The bathochromic shift of the π→π* band leads to an overlap with the n→π* excitation, making it more difficult to address, together with a diminishing thermal half-life. Both effects can be qualitatively reproduced and understood with the help of quantum mechanical calculations involving a combination of low-cost implicit solvation models and hybrid density functionals when including dispersion corrections.

## Supporting Information

File 1 General experimental information, synthetic procedures, UV–vis photochemistry and kinetic studies, computational methods, and X-ray crystallographic details.
