# Supplementary material for: Fluorinated azobenzenes as supramolecular halogen-bonding building blocks
Source: Beilstein J Org Chem. 2019 Aug 23;15:2013–9. doi: 10.3762/bjoc.15.197 (PMC6720338; doi:10.3762/bjoc.15.197)
Supplement: File 1 — General experimental information, synthetic procedures, UV–vis photochemistry and kinetic studies, computational methods, and X-ray crystallographic details. [file Beilstein_J_Org_Chem-15-2013-s001.pdf]

BEILSTEIN JOURNAL OF ORGANIC CHEMISTRY

Supporting Information

for

Fluorinated azobenzenes as supramolecular halogen-bonding building blocks

Esther Nieland, Oliver Weingart and Bernd M. Schmidt

Beilstein J. Org. Chem. **2019,** *15,* 2013–2019. doi:10.3762/bjoc.15.197

General experimental information, synthetic procedures, UV–vis photochemistry and kinetic studies, computational methods, and X-ray crystallographic details

License and Terms: This is a supporting information file under the terms of the Creative Commons Attribution License (http://creativecommons.org/licenses/by/4.0). Please note that the reuse, redistribution and reproduction in particular requires that the authors and source are credited.
The license is subject to the *Beilstein Journal of Organic Chemistry* terms and conditions: (https://www.beilstein-journals.org/bjoc)

Table of Contents

I. General S2

II. Experimental details S3

III. UV–vis spectroscopy.. S4

IV. Computational details.. S13

V. Crystallographic details S33

VI. References S36

I. General

Solvents and starting materials were purchased from Sigma-Aldrich, TCI, Fisher Scientific, Alfa Aesar or Carl Roth and used as received. Dry solvents were obtained from an MBRAUN solvent purification system. Reactions were monitored by thin-layer chromatography (TLC) carried out on silica gel plates (ALUGRAM® Xtra SIL G/UV254, Macherey Nagel) using UV light for visualization. Column chromatography was carried out with silica gel (Silica 60 M, 0.04–0.063 mm, Macherey Nagel) using eluents as specified.

UV–vis spectroscopy was performed on Cary 60 equipped with a Peltier thermostated cell holder. Quartz cuvettes and solvents of spectrometric grade were used. A 405 nm LED (M405L3) and a 455 nm LED (M455L3), together with a LED driver (LEDD1B), all from Thorlabs, were used for photoisomerisation.

II. Experimental details

2,2',6,6'-Tetrafluoro-4,4'-diiodoazobenzene (**A1**) was synthesized following a procedure by Hecht et al.,[1] while 4,4'-diiodooctafluoroazobenzene (**A2**) was synthesized according to Barrett et al.[2] The syntheses of 4,4'-(10-mesitylanthracene-1,8-diyl)bis(3,5-dimethylpyridine) (**U1**) and 4,4'-diiodoethynyloctafluoroazobenzene (**A3**) were described previously.[3]

III. UV–vis spectroscopy

Absorption spectra of **A1**, **A2** and **A3** were recorded at 298.15 K after irradiation with green light (λ_{irr} = 565 nm) at given times.

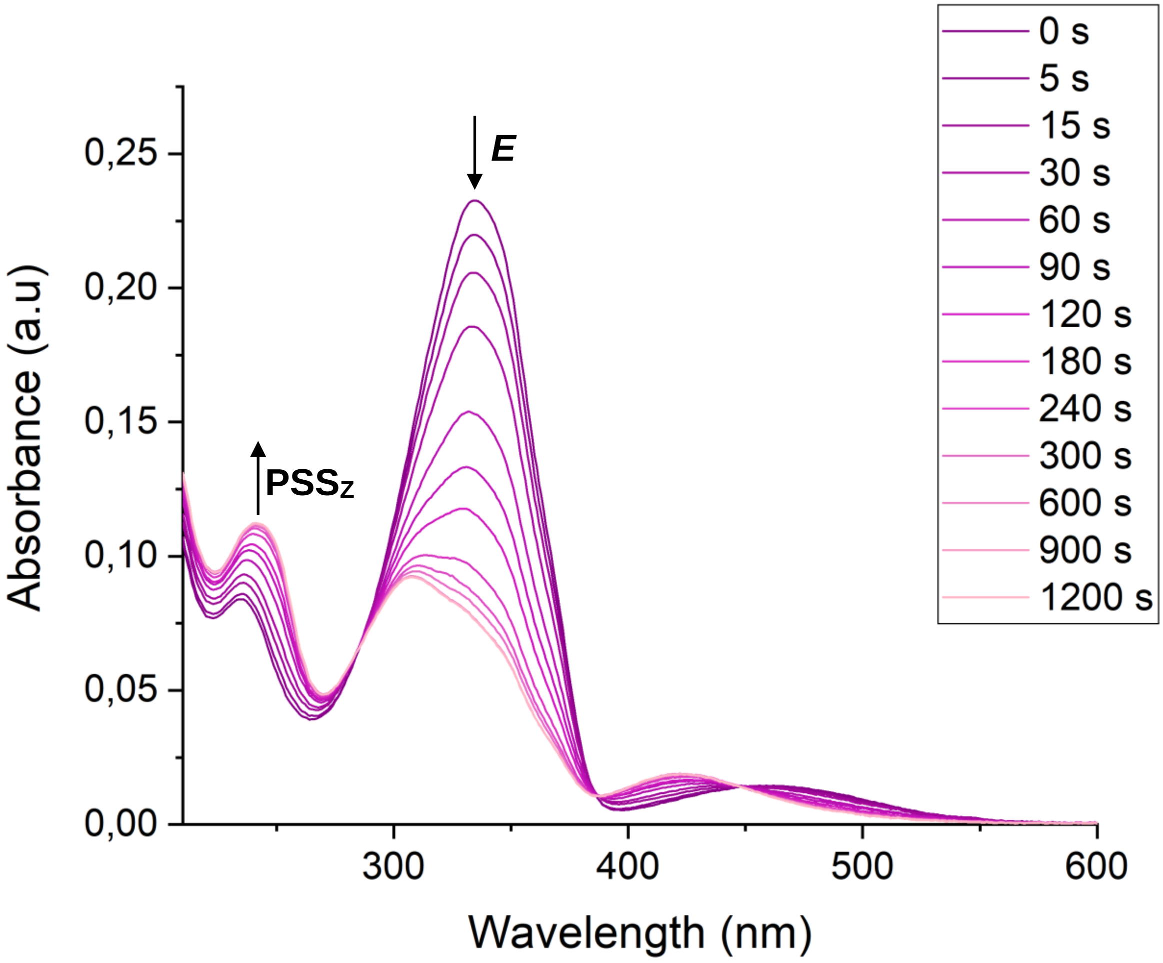

Figure S1. UV–vis spectra of **A1** in MeCN (*c* = 6.68 µmol/L) after irradiation.

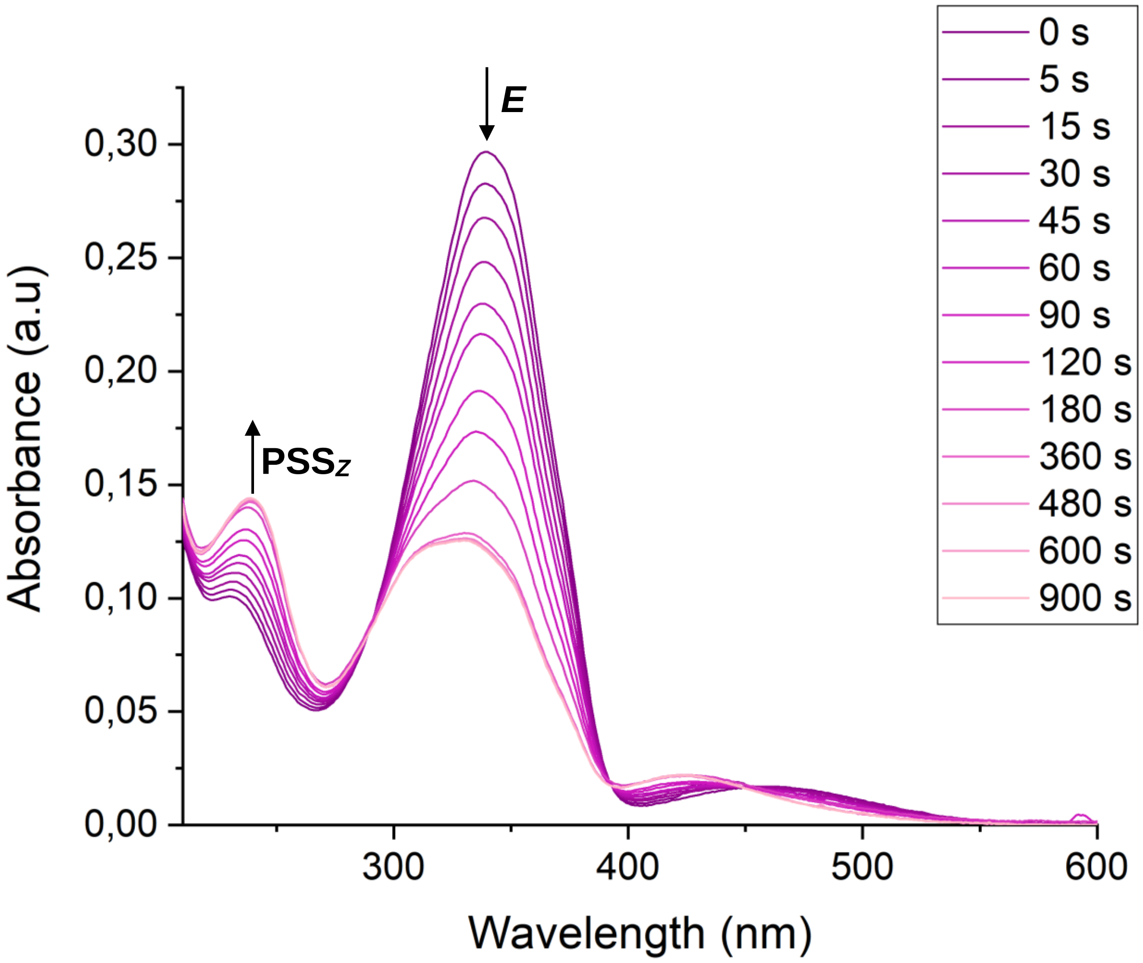

Figure S2. UV–vis spectra of **A2** in MeCN (c = 10.5 µmol/L) after irradiation.

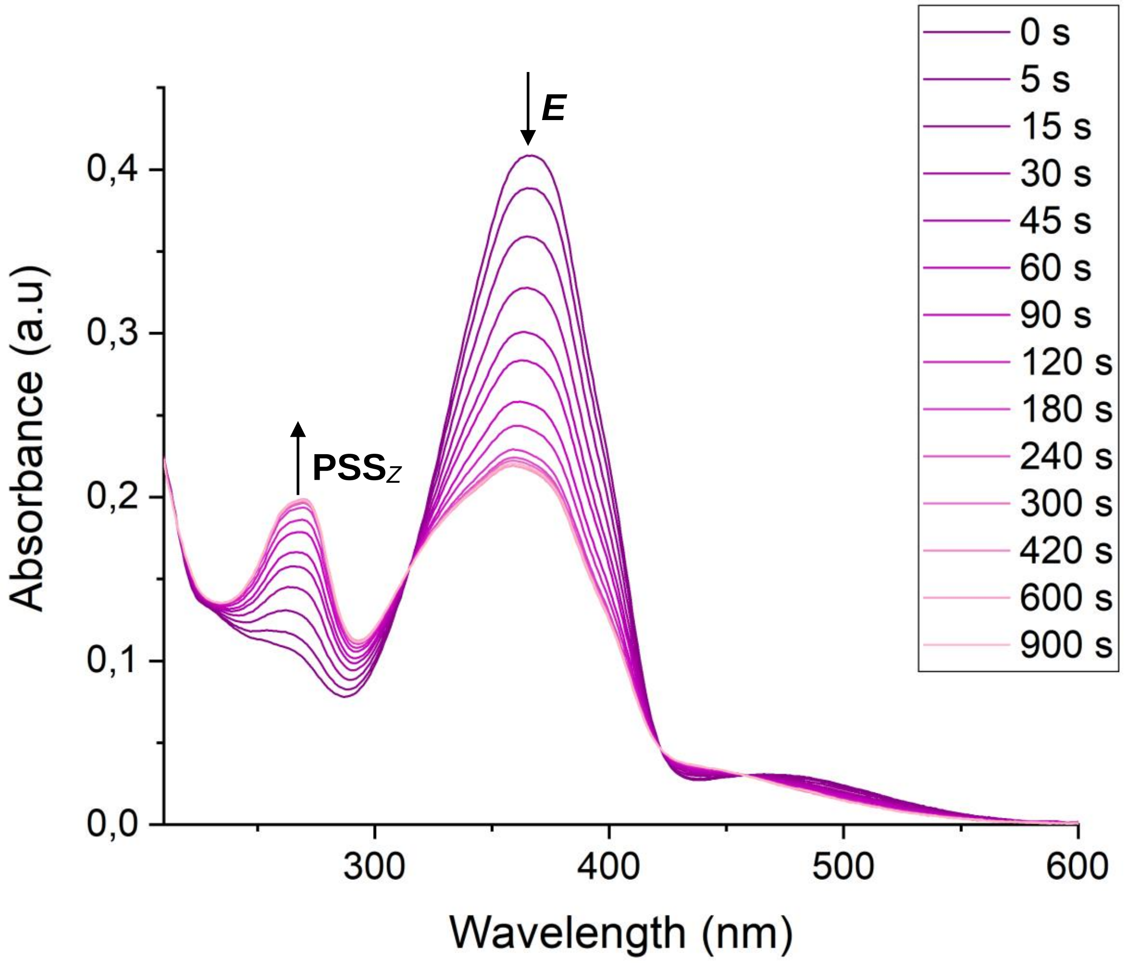

Figure S3. UV–vis spectra of **A3** in MeCN (c = 9.26 µmol/L) after irradiation.

Irradiation of **A2** and **A3** was also performed in DCM and cyclohexane. Peak positons were determined using the OriginPro Peak Analyzer tool.

Table S1. Spectral features of **A2** and **A3**.

	Solvent	$\lambda_{\pi-\pi^*}$ (E) (nm)	$\lambda_{n-\pi^*}$ (Z) (nm)	$\lambda_{n-\pi^*}$ (E) (nm)	Δ ($\lambda_{n-\pi^*}$) (nm)
**A2**	DCM	339	425	463	38
	MeCN	340	424	458	34
	CyHex	335	427	463	36
**A3**	DCM	367	n. d.	471	n. d.
	MeCN	364	n. d.	466	n. d.
	CyHex	359	436	472	36

The evolution of the absorbance at 345 nm (for **A1**) and 380 nm (for **A2** and **A3**) was observed at 60 °C and fitted to a monoexponential fit.

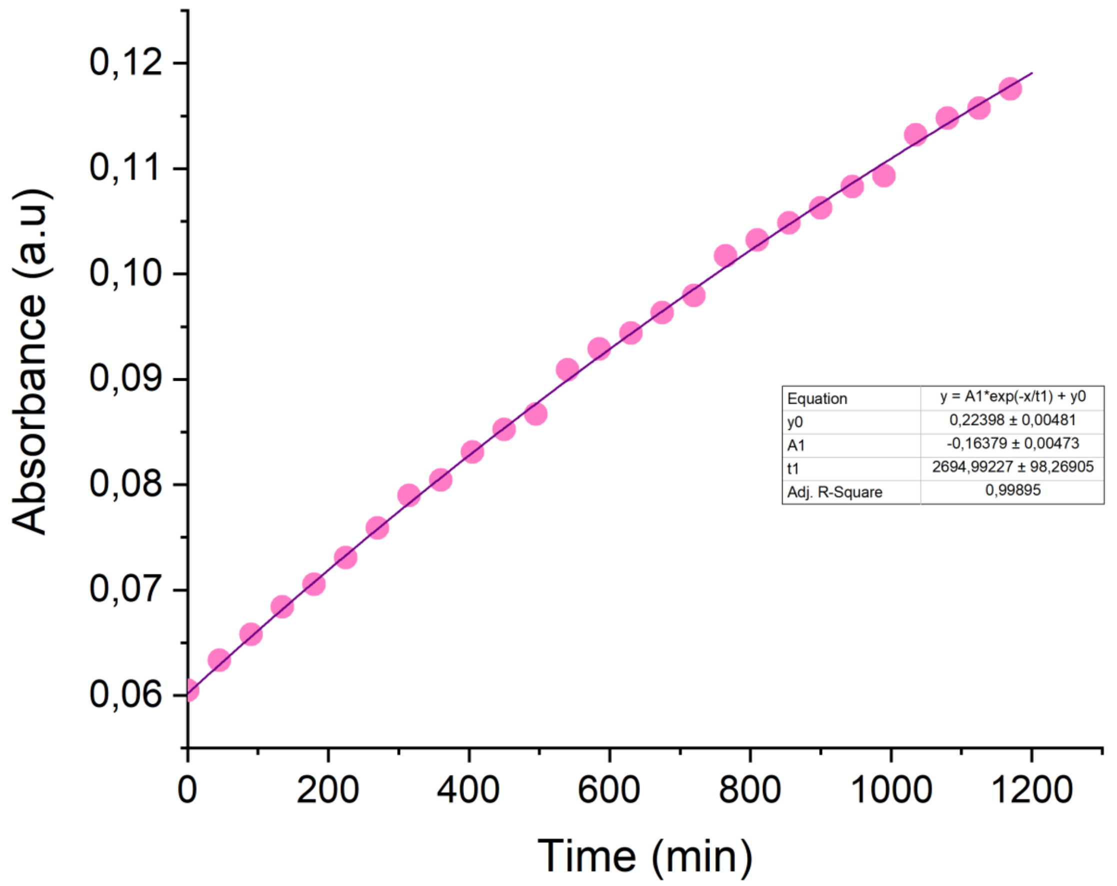

Figure S4. Thermal $E \rightarrow Z$ isomerization of **A1** in MeCN at 60 °C.

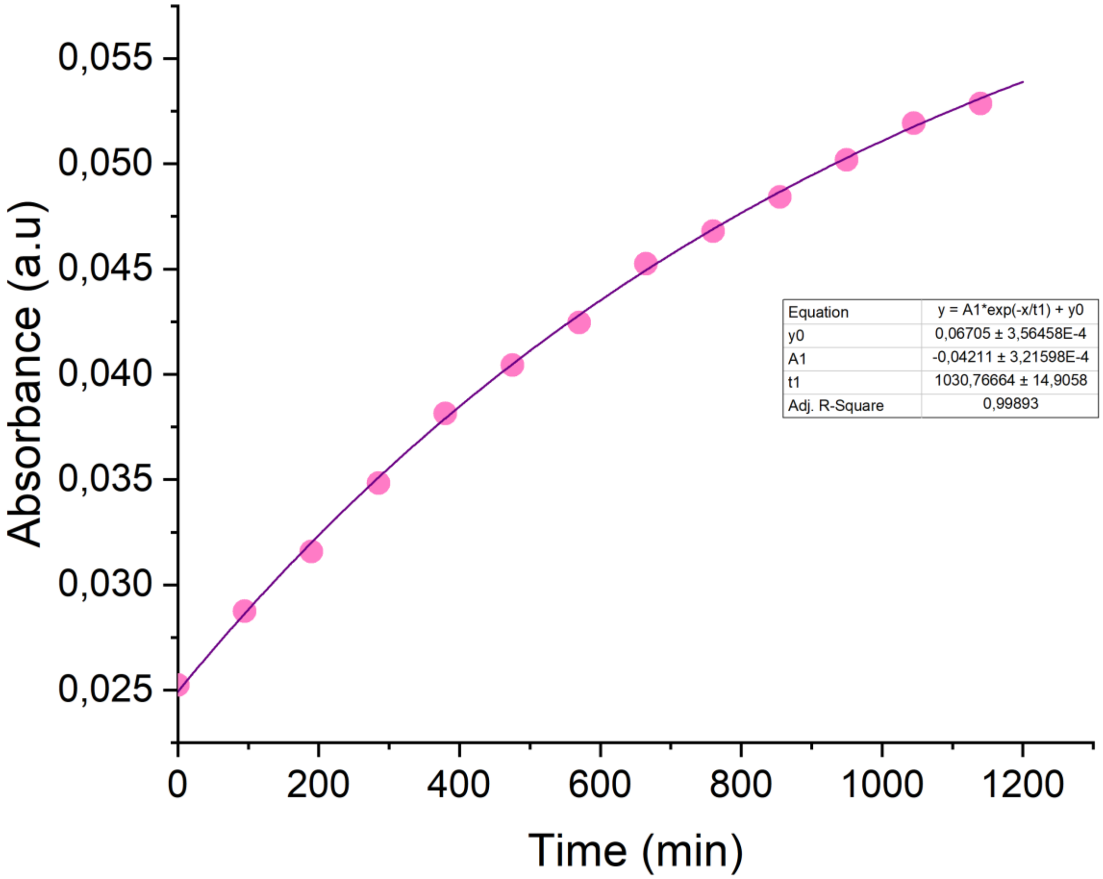

Figure S5. Thermal $E \rightarrow Z$ isomerization of **A2** in MeCN at 60 °C.

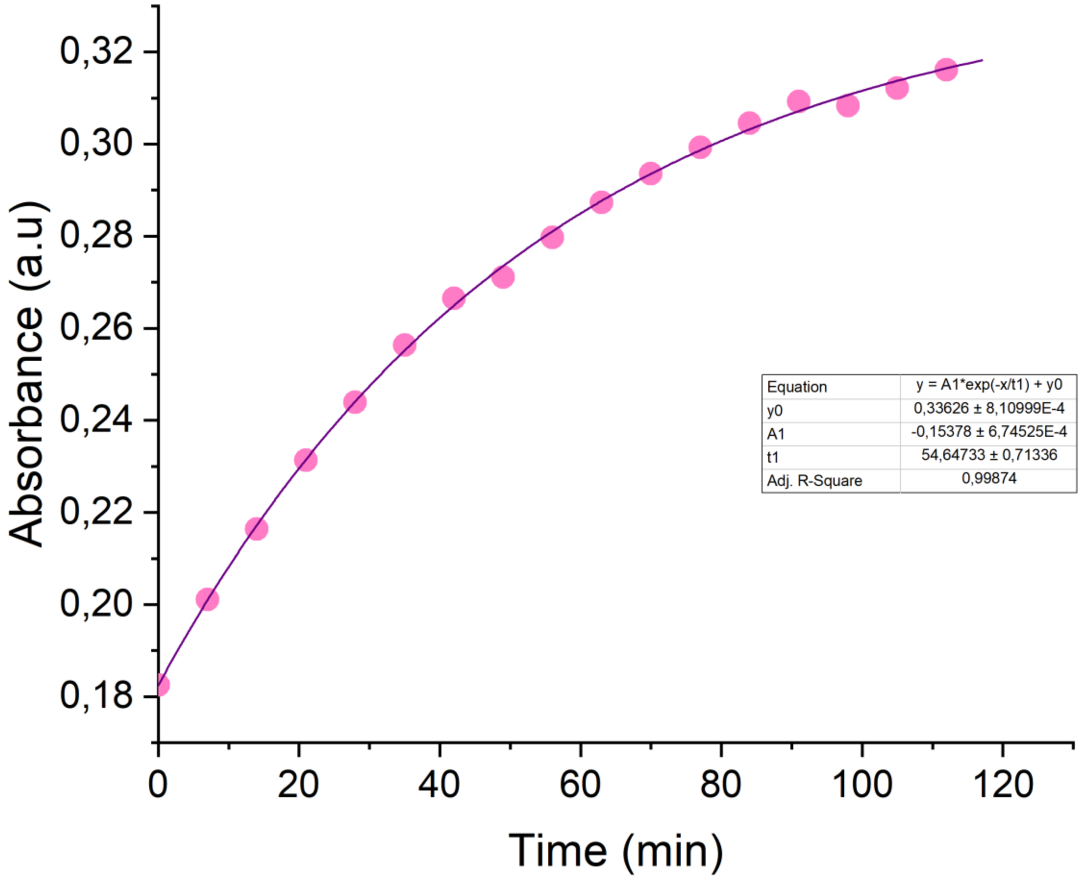

Figure S6. Thermal $E \rightarrow Z$ isomerization of **A3** in MeCN at 60 °C.

Table S2. Thermal stabilities of the *Z*-isomers at 60 °C in MeCN.

**Compound**	**A1**	**A2**	**A3**
$\tau_{1/2}$ (min)	2695	1030	55

The thermal stability of **A3** was determined at different temperatures.

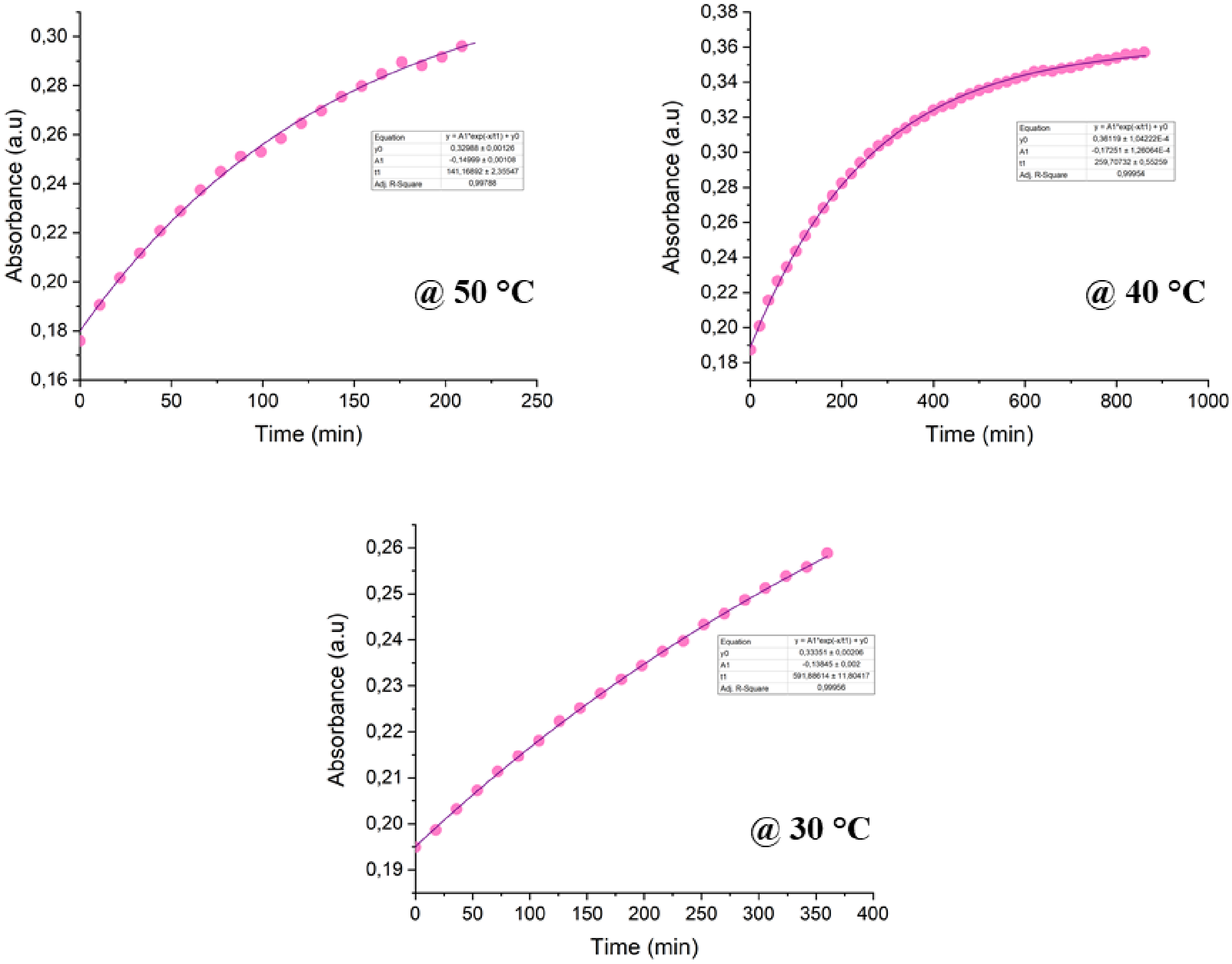

Figure S7. Thermal stability of **A3** at different temperatures in MeCN.

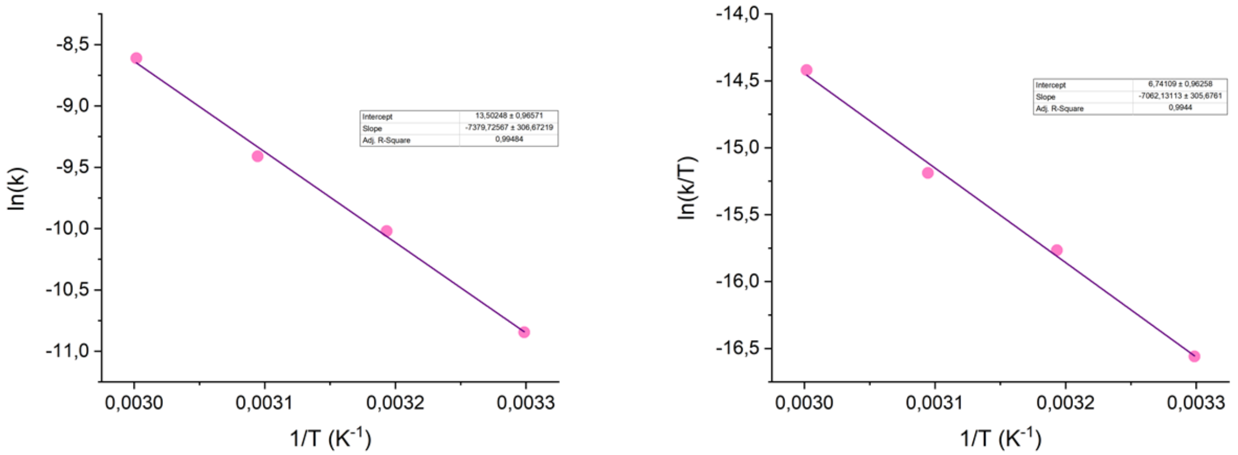

k (s^{-1})	$\tau_{1/2}$ (h)	A (s^{-1})	E_A (kJ/mol)	$\Delta H^‡$ (kJ/mol)	$\Delta S^‡$ (J/mol)	$\Delta G^‡$ (kJ/mol)
1.29×10^{-5}	14.98	7.31×10^{5}	61	59	-141	101

Figure S8. Arrhenius plot (left) and Van´t Hoff plot (right) for **A3**; the corresponding parameters were calculated at 298.15 K using the Arrhenius and Eyring equations.

IV. Computation details

Quantum mechanical calculations were performed by applying density functional theory and the polarizable continuum model[4] (PCM) for the description of solvent effects. All geometry optimizations were performed with the Gaussian 16 program.[5] For the computation of absorption properties, TD-DFT methods were used. The exact procedures are described in the corresponding sections.

IV.1 Method evaluation and benchmark

To find a suitable method for the computation of thermal *Z*→*E* isomerization barriers and corresponding half-lives in implicit PCM solvent, we first performed a series of benchmark calculations on fluorine-substituted azobenzenes (ABs). We tested a variety of functionals including pure, and hybrid approaches. The computed data were compared with values for fluoro-ABs in MeCN, measured at 60°C.[6] Structures of the investigated systems are given in Figure S9. Note that we have slightly adapted the naming convention of Hecht and co-workers,[6] to match the naming of the fluorine/iodine target systems, thus F2 corresponds to F4 in the original paper, F3 is F6 etc.

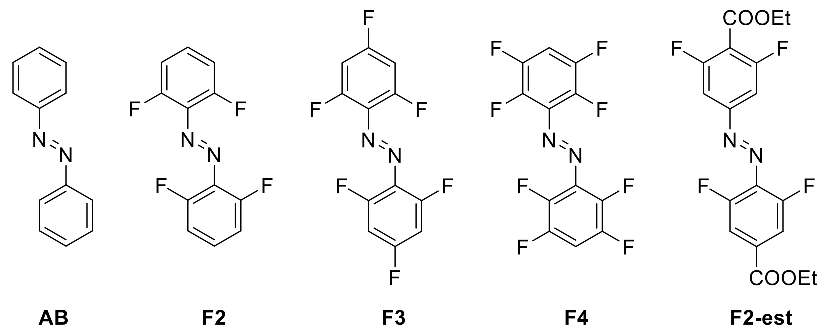

Figure S9. Azobenzene and fluorinated derivatives used for benchmarking.[6]

The transition state (TS) was modeled by performing constrained geometry optimizations along the CNN angle starting from the *Z*-isomer of the corresponding azobenzene. The point of highest energy along this coordinate was used as the initial point for TS optimizations with the B3LYP method, the 6-311G* basis set and MeCN as implicit solvent. The stationary nature of TSs, educts and products was identified through vibrational analyses, yielding one imaginary frequency for TSs (*380i-446i*) and only positive eigenvalues for *Z*- and *E*-structures. Intrinsic reaction coordinate (IRC) computations validated that the TS in fact leads to the corresponding *Z*-educt and *E*-product. The resulting geometries were then used as the starting point for structure optimizations with the tested functionals. The structures for *Z*-, *E*-isomers

and TS are very similar for the investigated systems. They are shown in Figure S10 by B3LYP/6-311G* optimized structures obtained for F2 as example. The optimized *E*-structures are not planar but significantly twisted among the CN axis and slightly distorted along CNNC dihedrals (20° and -177°).

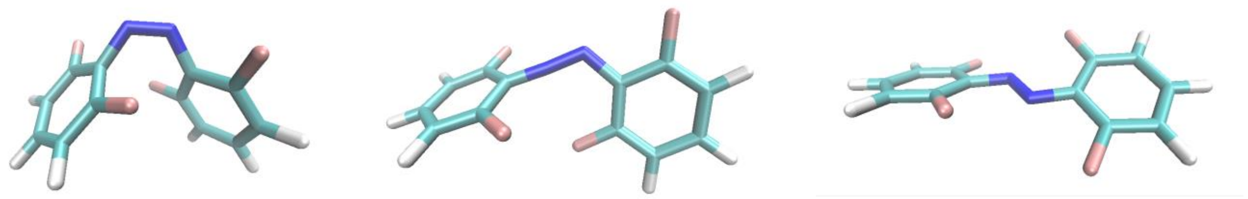

Figure S10. B3LYP-D3/6-311G* optimized structures of F2 in implicit MeCN. From left to right: *Z*-isomer, transition state and *E*-isomer.

The lowest energy transition points for all models has an almost planar CNN angle with nearly perpendicular arrangement of the phenyl rings (CCNN ≈ 70–90°). These geometries largely agree with findings from earlier computations of pure and substituted azobenzenes in vacuo and PCM solution reported, e.g., by Knie et al.,[6] Ritze et al.,[7] and Liu et al.[8] Thermal rate constants and half-lives were calculated using the KistHelp software package.[9] Computations involved conventional transition state theory (TST) considering also the effect of one-dimensional Wigner tunneling.[9]

Table S3. Half-lives $\tau_{1/2}$ (60°) [h] in benchmark sets using hybrid functionals. Experimental data from ref. [6].

**Model**	PBE0/ 6-311G*, D3	wB97XD/ 6-311G*	TPSSh/ 6-311G*, D3	B3LYP/ 6-311G*, D3	CAM-B3LYP/ 6-311G*	CAM-B3LYP/def2-TZVP, D3	$\tau_{1/2}$ (60°) exp.
**AB**	1.2	34.6	0.8	1.0	5.2	6.0	4
**F2**	622.3	6662.1	274.4	417.9	4334.5	511.1	92
**F3**	945.4	24336.8	444.8	451.3	3023.7	4059.9	95
**F4**	102.8	1362.6	63.7	68.3	437.8	54.6	27
**F2-est**	3.6	312.0	3.1	3.2	67.9	5.6	15

Table S4. Half-lives $\tau_{1/2}$ (60°) [h] in benchmark sets using pure functionals. Experimental data from ref. [6].

Model	BP86/ 6-311G*, D3	BLYP/ 6-311G*, D3	M06L/ 6-311G*, D3	B97D3/ 6-311G*	PBEPBE/ 6-31G*, D3	$\tau_{1/2}$ (60°) exp.
**AB**	0.03	0.01	0.16	0.11	0.02	4
**F2**	13.7	11.8	78.7	20.0	12.5	92
**F3**	19.3	11.9	92.8	27.3	13.6	95
**F4**	4.4	3.5	24.5	6.2	4.2	27
**F2-est**	0.11	0.17	0.38	0.08	0.2	15

As the target molecules in this study include heavy and large iodine atoms, dispersion corrections were applied for all methods in the test set, except for one CAM-B3LYP reference calculation including dispersion (see Table S3). Comparison with the data from Knie et al.,[6] who used the same set of molecules without including dispersion corrections, reveals that dispersion effectively increases the thermal barriers, thus leading to significantly longer half-lives (ca. 1–2 orders of magnitude). Long-range corrections, as, e.g., included in CAM-B3LYP or the wB97XD functionals tend to largely overshoot in our case. A similar study by Rietze et al.[7] indicates that this effect is likely an artifact caused by the PCM solvation, as it does not appear in gas-phase calculations. In further computations, we therefore did not include long-range corrections, neither for geometry optimizations nor for calculation of thermal barriers. For the computation of rate constants and thus half-lives we shall expect an accuracy of ca. 1 order of magnitude.[10] The hybrid functionals B3LYP, TPSSh and PBE0 all lie within this range and reflect the trend also found in the experiments, namely F3 > F2 > F4 > F2-est > AB, considering half-lives. The same trend is also found for the pure functionals, but with significantly shorter half-lives as in the hybrids. While the values for F2–F4 all lie within chemical accuracy, the times for AB and F2-est are underestimated in some cases. F4 half-lives are overestimated in the hybrids, and underestimated in all pure functionals. Apparently, none of the applied DFT methods yields a fully satisfying result that describes all systems on equal footing when PCM solvation is used. Overall, B3LYP is the only functional among the hybrids that delivers a suitable balance between F2 and F3, while TPSSh and PBE0 attribute a much larger relative half-life to F3. Among the pure functionals, M06L appears to be the most suitable in terms of balance, giving even the best match in absolute values for F2–F4. Based on these tests, we decided to use B3LYP-D3 and M06L-D3 approaches to compute the corresponding thermal data for the iodine/fluorine compounds in this study.

IV.2 Optimization of fluorine/iodine-azobenzenes

For structure optimization of the target systems **A1**–**3**, we adopted a similar strategy as described in the benchmark study. Geometries were optimized using B3LYP and M06L functionals with Grimme D3 dispersion corrections and the def2-TZVP basis set for H, C, N and F atoms. The all-electron basis DGDZVP was used to describe iodine. MeCN solvation was considered through the PCM model. Stationary points were characterized by vibrational analyses, yielding only positive eigenvalues for *Z*- and *E*-minima and one imaginary frequency for the TSs. Figure S11 gives an overview of all optimized structures and computed IRC paths. The geometries are nearly equivalent for B3LYP and M06L, therefore only M06L-optimized points are shown. The computed IRC paths stop somewhat before reaching the true minima, a consequence of PCM solvation and the flat potential energy surface in these regions. Linear interpolation towards the optimized geometries however reveal that further relaxation from there proceeds without any barrier. The barrier heights decrease in the order **A1** > **A2** > **A3**.

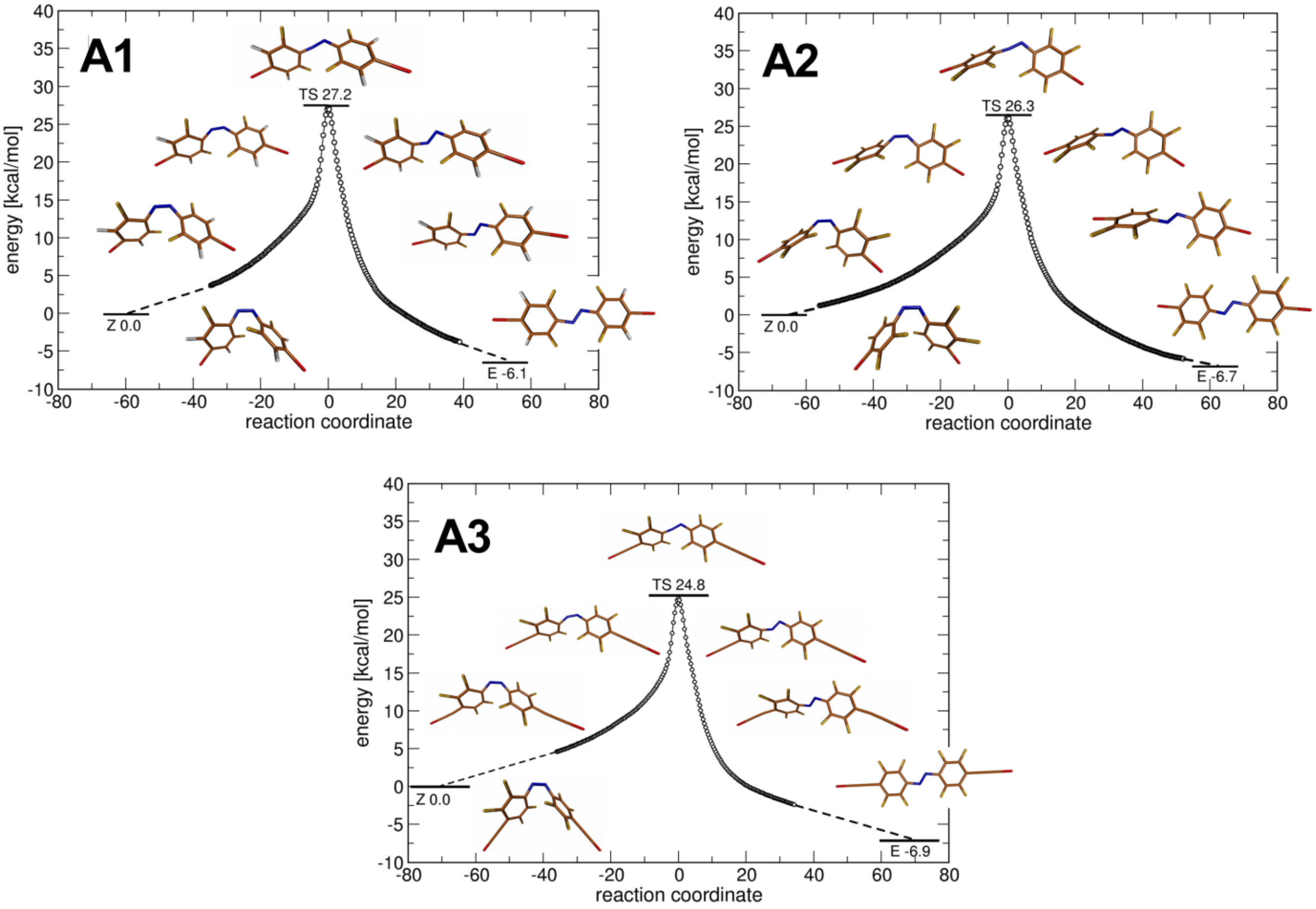

Figure S11. Minima, TS and structures along calculated IRC paths in **A1**, **A2** and **A3** computed with M06L-D3/def2-TZVP/DGDZVP. Electronic energies in kcal/mol are given relative to the **Z**-isomer.

Tables S5 and S6 report selected structural parameters for both methods. For the *E*-isomer, we find two structures in each model that are very close in energy. ($E_{\mathrm{diff}} < 0.04$ kcal/mol), see Table S6) E_1 corresponds to the optimized structure at the end of the IRC path. These structures are somewhat twisted along C–N bonds (ca. −10°, for M06L and up to −25° in B3LYP, see Table S5). The second minimum E_2 is almost planar (C–C–N=N dihedral ≈1.4–2.6°). *Z*-isomers are slightly twisted along the N=N bond (ca 13–15°), the phenyl rings are tilted (C–C–N=N dihedral ca. 50–53°). Both *Z*- and *E*-isomers have formal C_2 symmetry, while the TS is asymmetric with almost straight connection along C–N=N (≈175°) on one side and almost perpendicular arrangement of the phenyl rings (C–C–N=N ≈70–100°).

Table S5. Selected structural parameter in M06L computed F/I-ABs. The TS structures are asymmetric; the corresponding values for differing structural parameters are given in italics.

		**N=N [Å]**	**C-N [Å]**	**C-I [Å]**	**C-N=N [°]**	**C-C-N [°]**	**C-N=N-C [°]**	**C-C-N=N [°]**
**A1**	Z	1.248	1.412	2.101	122.51	123.96	13.2	53.0
	TS	1.227	1.418 *1.304*	2.100	118.22 *175.56*	127.85 123.37	218.9	-8.9 *-97.9*
	E_1	1.267	1.388	2.099	115.79	129.29	177.8	-10.4
	E_2	1.268	1.387	2.099	115.83	129.54	180.7	2.6
**A2**	Z	1.246	1.413	2.081	122.77	123.22	13.4	52.2
	TS	1.225	1.420 *1.303*	2.081 *2.078*	118.25 *175.83*	127.21 *122.56*	139.3	7.6 *100.5*
	E_1	1.266	1.388	2.078	115.51	128.53	181.9	10.0
	E_2	1.267	1.388	2.078	115.58	128.78	180.4	2.3
**A3**	Z	1.248	1.409	1.981	123.06	123.39	14.6	50.8
	TS	1.225	1.413 *1.299*	1.984 *1.979*	118.70 *175.57*	127.08 *121.32*	225.9	-5.6 *71.5*
	E_1	1.269	1.385	1.980	115.58	128.49	177.5	-10.2
	E_2	1.270	1.384	1.980	115.59	128.78	180.3	1.4

Table S6. Selected structural parameter in B3LYP computed F/I-ABs. The TS structures are asymmetric; the corresponding values for differing structural parameters are given in italics.

		N=N [Å]	C-N [Å]	C-I [Å]	C-N=N [°]	C-C-N [°]	C-N=N-C [°]	C-C-N=N [°]
**A1**	Z	1.241	1.423	2.119	122.59	124.04	9.9	56.4
	TS	1.220	1.424 *1.313*	2.121	118.71 *177.43*	127.71 *122.77*	199.3	-4.8 *-87.3*
	E_1	1.254	1.398	2.118	116.05	127.92	176.8	-24.1
	E_2	1.256	1.396	2.117	116.57	129.24	180.3	2.5
**A2**	Z	1.238	1.425	2.101	122.72	123.28	9.7	56.4
	TS	1.216	1.427 *1.312*	2.103 *2.098*	118.72 *177.51*	126.96 *122.03*	154.3	1.1 *99.8*
	E_1	1.254	1.398	2.099	116.03	127.75	182.3	17.7
	E_2	1.255	1.397	2.099	116.31	128.48	180.2	2.4
**A3**	Z	1.239	1.422	2.001	122.90	123.26	10.6	55.5
	TS	1.214	1.420 *1.* 309	2.005 *1.998*	119.31 *178.21*	126.81 *121.66*	191.2	-1.0 *80.5*
	E_1	1.256	1.395	2.000	116.18	127.89	177.6	15.5
	E_2	1.257	1.394	2.000	116.37	128.47	180.3	1.6

Table S7. Electronic energies of identified stationary points in F/I-substituted ABs wrt. lowest energy structure. Imaginary frequencies (cm^{-1}) are given for the TS.

		M06L	B3LYP
**A1**	Z	6.09	6.31
	TS	33.30 *403.5i*	34.72 *428.4i*
	E_1	0.01	0.00
	E_2	0.00	0.04
**A2**	Z	6.71	6.32
	TS	32.98 *394.5i*	34.72 *414.6i*
	E_1	0.01	0.00
	E_2	0.00	0.04
**A3**	Z	6.87	6.57
	TS	31.70 *373.9i*	33.61 *395.2i*
	E_1	0.00	0.00
	E_2	0.03	0.02

Table S8. Dipole moments (Debye) of isomers and transition states.

		M06L	B3LYP
**A1**	Z	4.4247	4.5211
	TS	4.1292	4.8508
	E_1	0.1606	0.2257
	E_2	0.0600	0.0267
**A2**	Z	3.3206	3.1377
	TS	4.1367	5.3263
	E_1	0.0941	0.1004
	E_2	0.0049	0.0077
**A3**	Z	5.5521	5.1273
	TS	6.4560	8.2042
	E_1	0.2269	0.1576
	E_2	0.0293	0.0181

IV.3 Computation of excited state absorption spectra

Absorption energies for B3LYP-D3 optimized geometries were computed using the time-dependent density functional approach (TDDFT) involving 20 roots, with the same basis set and solvent modelling used for optimization. The obtained vertical absorption energies and oscillator strengths were convoluted with a 0.2 eV Gaussian function (FWHM=0.2 eV). The resulting spectra are shown below. Table S9 reports excitation energies and oscillator strengths of the low-lying ππ* and nπ* states in comparison with experimentally obtained values in MeCN solution. Due to the mostly planar structures the nπ* vertical excitation in the *E*-isomers shows almost no oscillator strength, the corresponding excitation wavelengths are indicated in the UV spectra. The overall trend follows the experimental observations; we note, however, a red shift for all computed excitation energies wrt. to experimental values. Especially the nπ* excitation energies are largely underestimated. Considering long-range interactions with the CAM-B3LYP functional leads to better energies for the ππ* states in the E-isomer, but now these states are overestimated in the Z-isomer (Table S10).

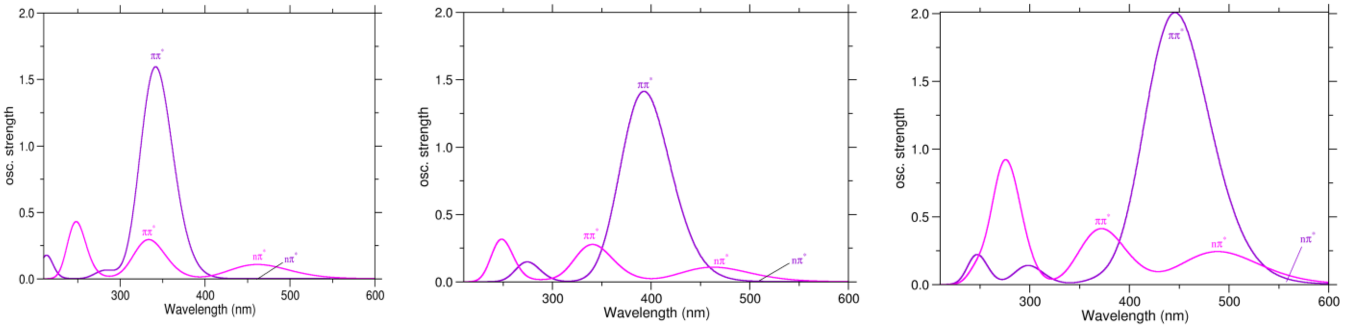

Figure S12. UV–vis absorption spectra computed with TD-B3LYP, obtained by convolution with a Gaussian function of 0.2 eV full-width half maximum (FWHM). The *E*-isomer is shown in violet, *Z* in magenta.

Table S9. Absorption data computed with TD-B3LYP-D3. Experimental values are shown in italics, computed oscillator strengths *f* are reported below the absorption values.

**Model**	***E*-ππ*** [nm]	***Z*-ππ*** [nm]	***E*-nπ*** [nm]	***Z*-nπ*** [nm]
**A1**	342 *332*	333 *307*	491 *432*	462 *423*
	f=1.596	*f=0.204*	*f=0.001*	*f=0.107*
**A2**	393 *340*	338 *330*	536 *458*	463 *424*
	f=1.389	*f=0.188*	*f=0.001*	*f=0.108*
**A3**	446 *364*	381 *359*	557 *466*	489 -
	f=2.0024	*f=0.1417*	*f=0.0019*	*f=0.2422*

Table S10. Absorption data computed with TD-CAM-B3LYP-D3. Experimental values are shown in italics, computed oscillator strengths *f* are reported below the absorption values.

**Model**	***E*-ππ*** [nm]	***Z*-ππ*** [nm]	***E*-nπ*** [nm]	***Z*-nπ*** [nm]
**A1**	387 *332*	280 *307*	531 *432*	439 *423*
	f=1.439	*f=0.099*	*f=0.001*	*f=0.061*
**A2**	342 *340*	280 *330*	498 *458*	439 *424*
	f=1.575	*f=0.484*	*f=0.001*	*f=0.108*
**A3**	380 *364*	307 *359*	508 *466*	452 -
	f=2.215	*f=0.615*	*f=0.001*	*f=0.120*

IV.4 Computation of electrostatic potentials

To obtain information on the halogen-bonding properties of the systems we computed electrostatic potential (ESP) maps at electron densities of 0.001 and 0.0001 with B3LYP/def2-TZVP and Grimme D3 dispersion corrections. The MoleCoolQt program[11] was used to visualize the results. ESP maps are shown in Figure S13, revealing strong positive potential values at the iodine atoms in the order **A3** > **A2** > **A1**.

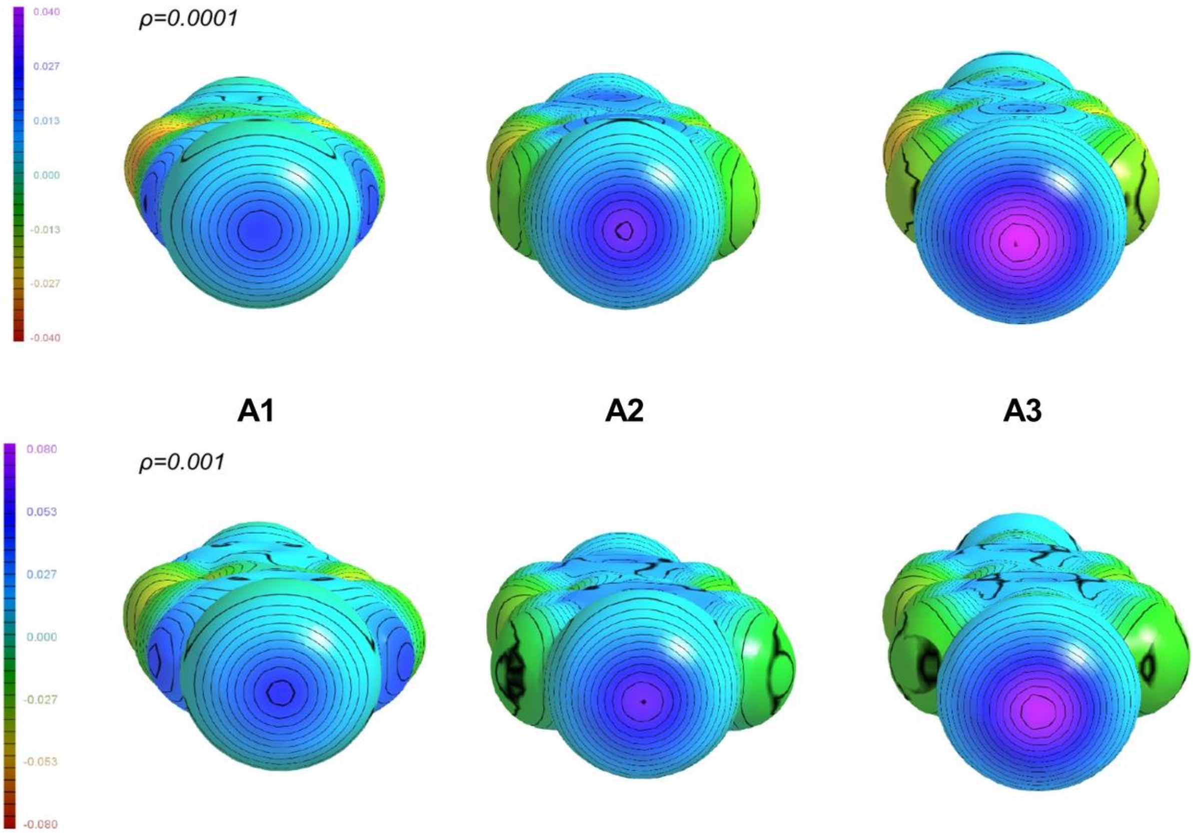

Figure S13. Map of the electrostatic potential plotted at different isodensity values of the electron density.

Figure S14 compares ESP maps in E- and Z-isomers, computed with M06L/def2-TZVP/DGDZVP.

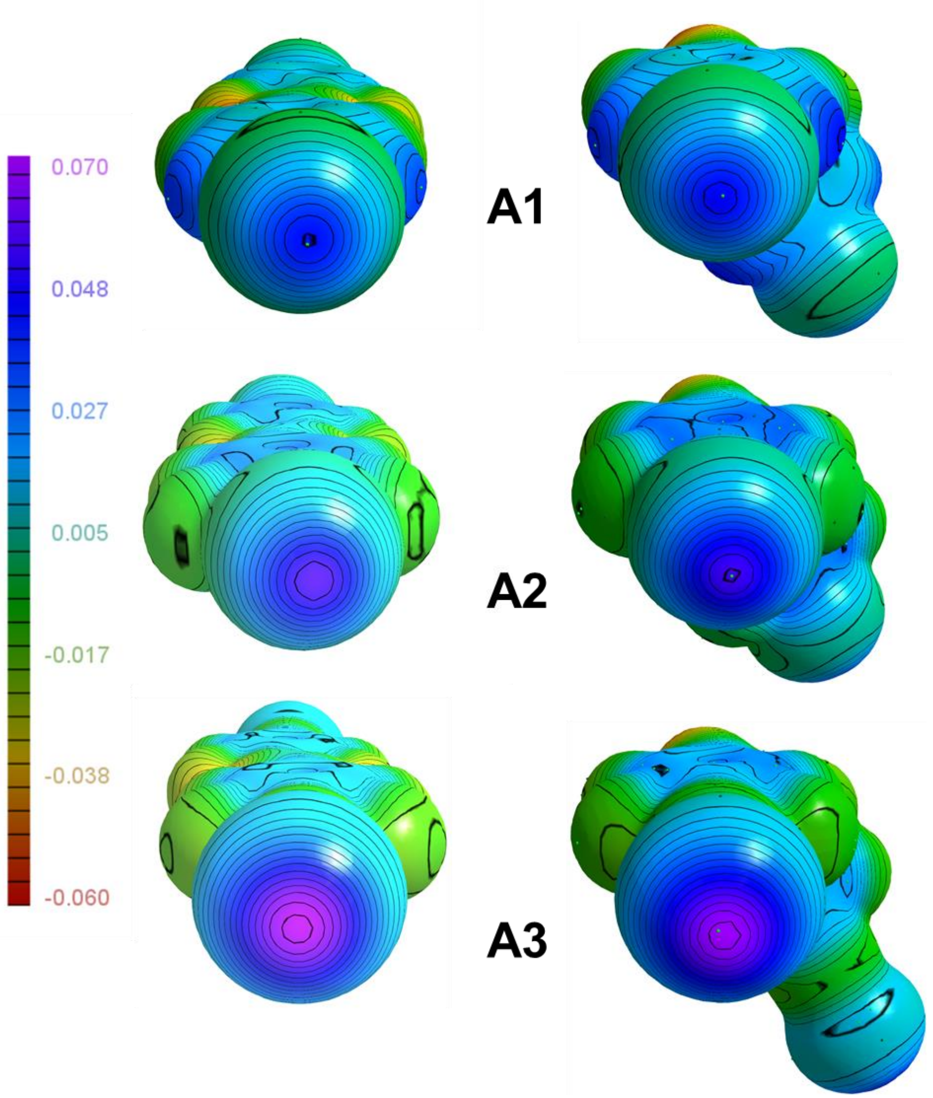

Figure S14. Comparison of electrostatic potentials in *Z*- and *E*-isomers of target systems **A1-3**. Values computed with M06L/def2-TZVP/DGDZVP, $\rho = 0.0001$.

IV.5 Calculation of thermodynamic data

The KistHelp program[9] was used to obtain thermodynamic properties of F/I- substituted ABs. A reaction path was modelled using the vibrational analyses of *Z*-isomers and the TS at experimental temperature (333.15 K), employing classical transition state theory (TST) and including Wigner tunneling.[9] Tables S11 and S12 show the resulting data for M06L and B3LYP methods in comparison with the experimental data. The computed half-lives follow the experimental trend, namely **A1** > **A2** > **A3**, with M06L obtained values underestimating the lifetimes by ca 1–2 orders of magnitude. B3LYP data are within chemical accuracy.

Table S11. Activation process parameters for the *Z-E* isomerization in MeCN at 60 °C, M06L, def2-TZVP basis for C, F, N, H, DGZVP all electron basis for iodine. Grimme D3 dispersion correction was applied.

Model	ΔU [kJ mol^{-1}]	ΔG [kJ mol^{-1}]	ΔH [kJ mol^{-1}]	ΔS [J mol^{-1}]	k_{Z-E} [s^{-1}]	$\tau_{1/2}$ [h]	$\tau_{1/2}$ exp. [h]
**A1**	113.82	105.93	108.87	8.84	1.9281 10^{-4}	1.0	32
**A2**	109.91	103.52	105.42	5.70	4.5815 10^{-4}	0.42	20
**A3**	103.89	97.13	99.34	6.64	4.5508 10^{-3}	0.04	1.8

Table S12. Activation process parameters *cis-trans* isomerization in MeCN at 60 °C, B3LYP, def2-TZVP basis for C, F, N, H, DGZVP all electron basis for iodine. Grimme D3 dispersion correction was applied.

Model	ΔU [kJ mol^{-1}]	ΔG [kJ mol^{-1}]	ΔH [kJ mol^{-1}]	ΔS [J mol^{-1}]	k_{Z-E} [s^{-1}]	$\tau_{1/2}$ [h]	$\tau_{1/2}$ exp. [h]
**A1**	124.10	114.32	119.06	14.22	9.4621 10^{-6}	20.35	32
**A2**	118.86	108.73	114.49	17.28	7.0619 10^{-5}	2.73	20
**A3**	113.13	99.89	108.69	26.42	1.6952 10^{-3}	0.11	1.8

IV.6 Cartesian coordinates of F/I-substituted ABs

***Z*-A1** M06L-D3/def2-TZVP	B3LYP-D3/def2-TZVP
24	24
Energy = -14807.9086338	Energy = -14808.6071465
C 0.628843 1.239176 2.003031	C 0.632691 1.238307 2.035851
N 0.335684 0.525988 3.186204	N 0.322507 0.529904 3.230111
N -0.335684 -0.525988 3.186204	N -0.322507 -0.529904 3.230111
C -0.628843 -1.239176 2.003031	C -0.632691 -1.238307 2.035851
C 0.335684 -1.654192 1.083644	C 0.322507 -1.681728 1.120058
C 0.052075 -2.504834 0.036390	C 0.012749 -2.502581 0.053349
C -1.251422 -2.954693 -0.119803	C -1.308846 -2.895919 -0.115379
C -2.248792 -2.581583 0.773050	C -2.299602 -2.495391 0.774837
C -1.912444 -1.753486 1.819692	C -1.936258 -1.692976 1.836400
C -0.335684 1.654192 1.083644	C -0.322507 1.681728 1.120058
C -0.052075 2.504834 0.036390	C -0.012749 2.502581 0.053349
C 1.251422 2.954693 -0.119803	C 1.308846 2.895919 -0.115379
C 2.248792 2.581583 0.773050	C 2.299602 2.495391 0.774837
C 1.912444 1.753486 1.819692	C 1.936258 1.692976 1.836400
F 1.598733 -1.258108 1.262215	F 1.606101 -1.329026 1.306918
H 0.849460 -2.800457 -0.628795	H 0.802883 -2.815449 -0.612507
I -1.721882 -4.225852 -1.724839	I -1.823370 -4.136570 -1.754514

H -3.270086 -2.916254 0.671188	H -3.333161 -2.786834 0.664299
F -2.855314 -1.377541 2.681634	F -2.879535 -1.295840 2.703746
F -1.598733 1.258108 1.262215	F -1.606101 1.329026 1.306918
H -0.849460 2.800457 -0.628795	H -0.802883 2.815449 -0.612507
I 1.721882 4.225852 -1.724839	I 1.823370 4.136570 -1.754514
H 3.270086 2.916254 0.671188	H 3.333161 2.786834 0.664299
F 2.855314 1.377541 2.681634	F 2.879535 1.295840 2.703746

**TS-A1** M06L-D3/def2-TZVP	B3LYP-D3/def2-TZVP
24	24
Energy = -14807.8652817	Energy = -14808.5598793
C -1.798461 1.268372 0.201407	C 1.803964 1.289071 -0.168419
N -0.643173 2.084368 0.302878	N 0.645648 2.111525 -0.266576
N 0.455470 1.552955 0.175283	N -0.453310 1.588231 -0.184334
C 1.656016 1.048956 0.113230	C -1.659389 1.072527 -0.121562
C 2.622543 1.452613 -0.841696	C -2.541916 1.318259 0.955764
C 3.873691 0.896016 -0.922577	C -3.794856 0.758047 1.043871
C 4.257282 -0.059145 0.015745	C -4.251320 -0.051126 0.006265
C 3.364425 -0.475396 0.999790	C -3.436768 -0.313637 -1.092241
C 2.092346 0.037365 1.007265	C -2.167810 0.215733 -1.125664
C -1.871724 -0.072323 -0.199984	C 1.859739 -0.085579 0.109895
C -3.063615 -0.749861 -0.320726	C 3.047039 -0.778542 0.209788
C -4.244901 -0.078160 -0.027882	C 4.238907 -0.083618 0.025775
C -4.234772 1.251122 0.374036	C 4.248079 1.278199 -0.251089
C -3.021233 1.893964 0.472788	C 3.036731 1.933466 -0.338615
F 2.240199 2.374018 -1.732336	F -2.085399 2.108393 1.950629
H 4.540918 1.236120 -1.701250	H -4.396959 0.977728 1.913511
I 6.191463 -0.875176 -0.052990	I -6.197005 -0.889847 0.100675
H 3.631253 -1.212552 1.743137	H -3.758037 -0.935754 -1.914981
F 1.214636 -0.356223 1.937804	F -1.358623 -0.037088 -2.176727
F -0.748787 -0.721881 -0.494017	F 0.720074 -0.763014 0.295557
H -3.051456 -1.781627 -0.639198	H 3.019986 -1.835617 0.428158
I -6.073718 -1.089570 -0.205072	I 6.074876 -1.123864 0.173908
H -5.139764 1.790868 0.608201	H 5.160682 1.835370 -0.398679
F -3.005729 3.165899 0.854200	F 3.044086 3.243897 -0.604398

**E1-A1** M06L-D3/def2-TZVP	B3LYP-D3/def2-TZVP
24	24
Energy = -14807.8652817	Energy = -14808.5598793
C -1.798461 1.268372 0.201407	C 1.803964 1.289071 -0.168419
N -0.643173 2.084368 0.302878	N 0.645648 2.111525 -0.266576
N 0.455470 1.552955 0.175283	N -0.453310 1.588231 -0.184334
C 1.656016 1.048956 0.113230	C -1.659389 1.072527 -0.121562
C 2.622543 1.452613 -0.841696	C -2.541916 1.318259 0.955764
C 3.873691 0.896016 -0.922577	C -3.794856 0.758047 1.043871
C 4.257282 -0.059145 0.015745	C -4.251320 -0.051126 0.006265
C 3.364425 -0.475396 0.999790	C -3.436768 -0.313637 -1.092241
C 2.092346 0.037365 1.007265	C -2.167810 0.215733 -1.125664
C -1.871724 -0.072323 -0.199984	C 1.859739 -0.085579 0.109895
C -3.063615 -0.749861 -0.320726	C 3.047039 -0.778542 0.209788
C -4.244901 -0.078160 -0.027882	C 4.238907 -0.083618 0.025775
C -4.234772 1.251122 0.374036	C 4.248079 1.278199 -0.251089
C -3.021233 1.893964 0.472788	C 3.036731 1.933466 -0.338615
F 2.240199 2.374018 -1.732336	F -2.085399 2.108393 1.950629
H 4.540918 1.236120 -1.701250	H -4.396959 0.977728 1.913511
I 6.191463 -0.875176 -0.052990	I -6.197005 -0.889847 0.100675
H 3.631253 -1.212552 1.743137	H -3.758037 -0.935754 -1.914981
F 1.214636 -0.356223 1.937804	F -1.358623 -0.037088 -2.176727
F -0.748787 -0.721881 -0.494017	F 0.720074 -0.763014 0.295557
H -3.051456 -1.781627 -0.639198	H 3.019986 -1.835617 0.428158
I -6.073718 -1.089570 -0.205072	I 6.074876 -1.123864 0.173908
H -5.139764 1.790868 0.608201	H 5.160682 1.835370 -0.398679
F -3.005729 3.165899 0.854200	F 3.044086 3.243897 -0.604398

**E2-A1** M06L-D3/def2-TZVP	B3LYP-D3/def2-TZVP
24	24
Energy = -14807.8652817	Energy = -14808.6164535
C -1.798461 1.268372 0.201407	C -0.244256 1.751860 0.008869
N -0.643173 2.084368 0.302878	N -0.500603 0.379305 0.012496
N 0.455470 1.552955 0.175283	N 0.500603 -0.379305 0.012496
C 1.656016 1.048956 0.113230	C 0.244256 -1.751860 0.008869
C 2.622543 1.452613 -0.841696	C -0.991598 -2.427967 -0.042536

C 3.873691 0.896016 -0.922577	C -1.092176 -3.804893 -0.047892
C 4.257282 -0.059145 0.015745	C 0.070071 -4.565635 0.000367
C 3.364425 -0.475396 0.999790	C 1.319918 -3.962714 0.051709
C 2.092346 0.037365 1.007265	C 1.377311 -2.584227 0.053465
C -1.871724 -0.072323 -0.199984	C 0.991598 2.427967 -0.042536
C -3.063615 -0.749861 -0.320726	C 1.092176 3.804893 -0.047892
C -4.244901 -0.078160 -0.027882	C -0.070071 4.565635 0.000367
C -4.234772 1.251122 0.374036	C -1.319918 3.962714 0.051709
C -3.021233 1.893964 0.472788	C -1.377311 2.584227 0.053465
F 2.240199 2.374018 -1.732336	F -2.134085 -1.735706 -0.094836
H 4.540918 1.236120 -1.701250	H -2.074293 -4.251605 -0.090071
I 6.191463 -0.875176 -0.052990	I -0.070071 -6.678134 -0.005716
H 3.631253 -1.212552 1.743137	H 2.238812 -4.527668 0.089654
F 1.214636 -0.356223 1.937804	F 2.587513 -2.011877 0.102833
F -0.748787 -0.721881 -0.494017	F 2.134085 1.735706 -0.094836
H -3.051456 -1.781627 -0.639198	H 2.074293 4.251605 -0.090071
I -6.073718 -1.089570 -0.205072	I 0.070071 6.678134 -0.005716
H -5.139764 1.790868 0.608201	H -2.238812 4.527668 0.089654
F -3.005729 3.165899 0.854200	F -2.587513 2.011877 0.102833

**Z-A2** M06L-D3/def2-TZVP	B3LYP-D3/def2-TZVP
24	24
Energy = -15204.9421173	Energy = -15205.6872855
C -0.634803 -1.241887 1.994174	C 0.073759 -1.391021 1.946809
N -0.337169 -0.523859 3.174494	N -0.012133 -0.619063 3.141040
N 0.337169 0.523859 3.174494	N 0.012133 0.619063 3.141040
C 0.634803 1.241887 1.994174	C -0.073759 1.391021 1.946809
C -0.337169 1.644538 1.079560	C -1.126591 1.282618 1.040229
C -0.028710 2.511483 0.048035	C -1.250282 2.167503 -0.018093
C 1.262219 2.989760 -0.126746	C -0.322851 3.182505 -0.215599
C 2.233113 2.591541 0.784431	C 0.723444 3.298665 0.693393
C 1.921120 1.751739 1.833993	C 0.835695 2.429856 1.763738
C 0.337169 -1.644538 1.079560	C 1.126591 -1.282618 1.040229
C 0.028710 -2.511483 0.048035	C 1.250282 -2.167503 -0.018093
C -1.262219 -2.989760 -0.126746	C 0.322851 -3.182505 -0.215599
C -2.233113 -2.591541 0.784431	C -0.723444 -3.298665 0.693393

C -1.921120 -1.751739 1.833993	C -0.835695 -2.429856 1.763738
F -1.599854 1.251954 1.228479	F -2.061253 0.339943 1.206831
F -1.002381 2.877701 -0.776662	F -2.287283 2.016354 -0.846271
I 1.726454 4.271696 -1.698438	I -0.498145 4.507279 -1.836592
F 3.486299 3.010589 0.660131	F 1.648870 4.251028 0.552205
F 2.875079 1.382869 2.678613	F 1.855067 2.567832 2.614431
F 1.599854 -1.251954 1.228479	F 2.061253 -0.339943 1.206831
F 1.002381 -2.877701 -0.776662	F 2.287283 -2.016354 -0.846271
I -1.726454 -4.271696 -1.698438	I 0.498145 -4.507279 -1.836592
F -3.486299 -3.010589 0.660131	F -1.648870 -4.251028 0.552205
F -2.875079 -1.382869 2.678613	F -1.855067 -2.567832 2.614431

**TS-A2** M06L-D3/def2-TZVP	B3LYP-D3/def2-TZVP
24	24
Energy = -15204.9002564	Energy = -15205.6420145
C 1.805043 1.248874 0.278543	C -1.325564 0.084856 1.860937
N 0.652674 2.060347 0.455257	N -0.080736 0.249539 1.183335
N -0.447801 1.547088 0.295395	N -0.065342 0.198022 -0.031307
C -1.648206 1.050159 0.200936	C -0.001739 0.174896 -1.341372
C -2.601658 1.532787 -0.727740	C 0.536330 -0.924208 -2.045747
C -3.851013 0.967850 -0.826426	C 0.572949 -0.941620 -3.423431
C -4.272504 -0.044282 0.032453	C 0.124774 0.132521 -4.188043
C -3.355477 -0.504571 0.973803	C -0.393392 1.226230 -3.499702
C -2.071506 -0.017105 1.031348	C -0.482598 1.247261 -2.124503
C 1.850052 -0.062572 -0.205999	C -2.576113 -0.157626 1.278204
C 3.053310 -0.716728 -0.372665	C -3.705219 -0.312103 2.060158
C 4.259250 -0.098202 -0.061353	C -3.635244 -0.231920 3.448551
C 4.222299 1.204371 0.417676	C -2.398174 0.009104 4.033301
C 3.022044 1.867312 0.578854	C -1.263223 0.164097 3.255949
F -2.243523 2.513656 -1.553937	F 0.987571 -1.975368 -1.340442
F -4.681120 1.448793 -1.750279	F 1.080558 -2.031811 -4.015260
I -6.194173 -0.833309 -0.085533	I 0.227903 0.104599 -6.288203
F -3.694876 -1.474861 1.820690	F -0.849489 2.295565 -4.166636
F -1.209678 -0.497514 1.927171	F -1.009668 2.310323 -1.492782
F 0.736438 -0.705296 -0.529458	F -2.705679 -0.244681 -0.045347
F 3.038100 -1.959510 -0.838698	F -4.870439 -0.540568 1.450366

I	6.064918	-1.096051	-0.307183	I	-5.353995	-0.470566	4.627080
F	5.344085	1.838529	0.734191	F	-2.274148	0.097829	5.359051
F	3.037973	3.106940	1.039520	F	-0.101934	0.393162	3.862332

**E1-A2** M06L-D3/def2-TZVP				B3LYP-D3/def2-TZVP			
24				24			
Energy = -15204.9002564				Energy = -15205.6973505			
C	1.805043	1.248874	0.278543	C	-0.507694	-0.144211	1.702984
N	0.652674	2.060347	0.455257	N	0.337183	-0.179595	0.589471
N	-0.447801	1.547088	0.295395	N	-0.061645	0.441885	-0.423854
C	-1.648206	1.050159	0.200936	C	0.759444	0.365742	-1.553027
C	-2.601658	1.532787	-0.727740	C	1.776979	-0.567538	-1.815856
C	-3.851013	0.967850	-0.826426	C	2.471734	-0.545367	-3.013190
C	-4.272504	-0.044282	0.032453	C	2.202201	0.401905	-3.994613
C	-3.355477	-0.504571	0.973803	C	1.196251	1.326851	-3.747247
C	-2.071506	-0.017105	1.031348	C	0.486507	1.303104	-2.560411
C	1.850052	-0.062572	-0.205999	C	-1.870832	0.196483	1.738506
C	3.053310	-0.716728	-0.372665	C	-2.590618	0.131388	2.919366
C	4.259250	-0.098202	-0.061353	C	-1.996671	-0.259582	4.114170
C	4.222299	1.204371	0.417676	C	-0.651010	-0.602003	4.090261
C	3.022044	1.867312	0.578854	C	0.072506	-0.554680	2.912470
F	-2.243523	2.513656	-1.553937	F	2.082322	-1.527841	-0.945376
F	-4.681120	1.448793	-1.750279	F	3.418152	-1.469947	-3.205866
I	-6.194173	-0.833309	-0.085533	I	3.280244	0.431904	-5.795637
F	-3.694876	-1.474861	1.820690	F	0.889526	2.262505	-4.650768
F	-1.209678	-0.497514	1.927171	F	-0.469417	2.214013	-2.377873
F	0.736438	-0.705296	-0.529458	F	-2.527760	0.553875	0.636755
F	3.038100	-1.959510	-0.838698	F	-3.887243	0.455449	2.881710
I	6.064918	-1.096051	-0.307183	I	-3.099910	-0.332659	5.898633
F	5.344085	1.838529	0.734191	F	-0.022557	-0.985938	5.205384
F	3.037973	3.106940	1.039520	F	1.360513	-0.896153	2.948360

**E2-A2** M06L-D3/def2-TZVP				B3LYP-D3/def2-TZVP			
24				24			
Energy = -15204.9002564				Energy = -15205.6972810			
C	1.805043	1.248874	0.278543	C	1.236849	1.261836	0.001591

N 0.652674 2.060347 0.455257	N -0.007699 0.627408 0.003941
N -0.447801 1.547088 0.295395	N 0.007699 -0.627408 0.003941
C -1.648206 1.050159 0.200936	C -1.236849 -1.261836 0.001591
C -2.601658 1.532787 -0.727740	C -2.516616 -0.678968 -0.046633
C -3.851013 0.967850 -0.826426	C -3.657426 -1.462983 -0.047548
C -4.272504 -0.044282 0.032453	C -3.589757 -2.851029 0.000634
C -3.355477 -0.504571 0.973803	C -2.332821 -3.438850 0.047231
C -2.071506 -0.017105 1.031348	C -1.186740 -2.664517 0.046323
C 1.850052 -0.062572 -0.205999	C 2.516616 0.678968 -0.046633
C 3.053310 -0.716728 -0.372665	C 3.657426 1.462983 -0.047548
C 4.259250 -0.098202 -0.061353	C 3.589757 2.851029 0.000634
C 4.222299 1.204371 0.417676	C 2.332821 3.438850 0.047231
C 3.022044 1.867312 0.578854	C 1.186740 2.664517 0.046323
F -2.243523 2.513656 -1.553937	F -2.682155 0.640593 -0.098862
F -4.681120 1.448793 -1.750279	F -4.842078 -0.844729 -0.096656
I -6.194173 -0.833309 -0.085533	I -5.332584 -4.020732 0.003016
F -3.694876 -1.474861 1.820690	F -2.200053 -4.767839 0.094438
F -1.209678 -0.497514 1.927171	F -0.008890 -3.286373 0.092340
F 0.736438 -0.705296 -0.529458	F 2.682155 -0.640593 -0.098862
F 3.038100 -1.959510 -0.838698	F 4.842078 0.844729 -0.096656
I 6.064918 -1.096051 -0.307183	I 5.332584 4.020732 0.003016
F 5.344085 1.838529 0.734191	F 2.200053 4.767839 0.094438
F 3.037973 3.106940 1.039520	F 0.008890 3.286373 0.092340

**Z-A3** M06L-D3/def2-TZVP	B3LYP-D3/def2-TZVP
28<br>Energy = -15357.2831136<br>C -0.980050 -1.000380 2.800050<br>N -0.481882 -0.396119 3.971391<br>N 0.481882 0.396119 3.971391<br>C 0.980050 1.000380 2.800050<br>C 0.172718 1.669289 1.877091<br>C 0.727717 2.419588 0.861479<br>C 2.112899 2.522446 0.695906<br>C 2.914321 1.848167 1.626274<br>C 2.362937 1.126578 2.660719	28<br>Energy = -15358.0414882<br>C -0.924386 -1.046459 2.836044<br>N -0.445581 -0.430609 4.024549<br>N 0.445581 0.430609 4.024549<br>C 0.924386 1.046459 2.836044<br>C 0.094278 1.704718 1.926717<br>C 0.625628 2.431999 0.878794<br>C 2.007087 2.528805 0.679678<br>C 2.831141 1.871824 1.602786<br>C 2.303811 1.165181 2.664193

C	-0.172718	-1.669289	1.877091	C	-0.094278	-1.704718	1.926717
C	-0.727717	-2.419588	0.861479	C	-0.625628	-2.431999	0.878794
C	-2.112899	-2.522446	0.695906	C	-2.007087	-2.528805	0.679678
C	-2.914321	-1.848167	1.626274	C	-2.831141	-1.871824	1.602786
C	-2.362937	-1.126578	2.660719	C	-2.303811	-1.165181	2.664193
F	-1.151409	1.651400	2.009706	F	-1.233747	1.673369	2.086796
F	-0.079552	3.057462	0.024534	F	-0.206714	3.053031	0.040185
C	2.674192	3.271200	-0.351350	C	2.546872	3.259505	-0.401717
F	4.234204	1.902413	1.510401	F	4.156561	1.925680	1.456367
F	3.158479	0.495846	3.514701	F	3.125810	0.552019	3.518987
F	1.151409	-1.651400	2.009706	F	1.233747	-1.673369	2.086796
F	0.079552	-3.057462	0.024534	F	0.206714	-3.053031	0.040185
C	-2.674192	-3.271200	-0.351350	C	-2.546872	-3.259505	-0.401717
F	-4.234204	-1.902413	1.510401	F	-4.156561	-1.925680	1.456367
F	-3.158479	-0.495846	3.514701	F	-3.125810	-0.552019	3.518987
C	-3.158479	-3.915698	-1.254753	C	-3.007433	-3.883177	-1.326133
I	-3.951249	-4.969933	-2.732572	I	-3.771334	-4.917685	-2.859148
C	3.158479	3.915698	-1.254753	C	3.007433	3.883177	-1.326133
I	3.951249	4.969933	-2.732572	I	3.771334	4.917685	-2.859148

**TS-A3** M06L-D3/def2-TZVP				B3LYP-D3/def2-TZVP			
28				28			
Energy = -15357.2435438				Energy = -15357.9983985			
C	1.642518	1.520426	0.116804	C	-1.652407	1.563519	0.445376
N	0.452536	2.039450	0.167407	N	-0.460775	2.072512	0.630604
N	-0.640037	2.578828	0.289987	N	0.628087	2.578271	0.808972
C	-1.802731	1.779359	0.220616	C	1.797983	1.848106	0.469019
C	-1.872086	0.413217	-0.085438	C	1.854905	0.546811	-0.054174
C	-3.084926	-0.231147	-0.171446	C	3.066364	-0.046717	-0.336845
C	-4.294724	0.443924	0.044243	C	4.280677	0.620397	-0.115710
C	-4.219670	1.807803	0.349739	C	4.217845	1.917362	0.406683
C	-3.009064	2.459427	0.431274	C	3.008147	2.518474	0.693704
C	2.083184	0.587742	1.092708	C	-2.340945	1.712466	-0.781121
C	3.355931	0.081608	1.070917	C	-3.593389	1.178014	-0.960885
C	4.264261	0.373162	0.039411	C	-4.259428	0.451577	0.038851
C	3.806895	1.255098	-0.954616	C	-3.562769	0.298874	1.248058

C 2.569528 1.841179 -0.908179	C -2.311716 0.826283 1.456329
F -0.767857 -0.286096 -0.306325	F 0.737681 -0.140591 -0.285649
F -3.107496 -1.524305 -0.462888	F 3.080895 -1.284507 -0.832428
C -5.526534 -0.220514 -0.042655	C 5.515370 0.006913 -0.409315
F -5.334954 2.489787 0.568767	F 5.345739 2.590864 0.633392
F -2.995850 3.748781 0.723424	F 3.006716 3.752129 1.188375
F 1.242369 0.265252 2.075613	F -1.735464 2.394991 -1.769657
F 3.723655 -0.754603 2.038709	F -4.192123 1.359982 -2.145340
C 5.549443 -0.182865 0.003359	C -5.546946 -0.089715 -0.158900
F 4.619437 1.570747 -1.960314	F -4.131366 -0.388258 2.247591
F 2.187377 2.687289 -1.863415	F -1.677475 0.651005 2.629632
C 6.661955 -0.665119 -0.028158	C -6.649547 -0.553478 -0.328632
I 8.480438 -1.455920 -0.080934	I -8.479371 -1.323139 -0.610432
C -6.590680 -0.794369 -0.116810	C 6.572534 -0.518045 -0.661444
I -8.329257 -1.731121 -0.237830	I 8.323336 -1.386362 -1.078910

**E1-A3** M06L-D3/def2-TZVP	B3LYP-D3/def2-TZVP
28	28
Energy = -15357.2435438	Energy = -15358.0519506
C 1.642518 1.520426 0.116804	C -1.751726 0.461881 -0.120890
N 0.452536 2.039450 0.167407	N -0.387157 0.393245 -0.400265
N -0.640037 2.578828 0.289987	N 0.387880 0.647110 0.555549
C -1.802731 1.779359 0.220616	C 1.751738 0.527661 0.290186
C -1.872086 0.413217 -0.085438	C 2.367575 -0.118128 -0.799800
C -3.084926 -0.231147 -0.171446	C 3.743069 -0.193252 -0.894117
C -4.294724 0.443924 0.044243	C 4.588755 0.370387 0.069703
C -4.219670 1.807803 0.349739	C 3.976283 1.008914 1.153229
C -3.009064 2.459427 0.431274	C 2.602999 1.077858 1.264486
C 2.083184 0.587742 1.092708	C -2.595502 0.453702 -1.245432
C 3.355931 0.081608 1.070917	C -3.969293 0.486616 -1.124304
C 4.264261 0.373162 0.039411	C -4.590218 0.512384 0.128949
C 3.806895 1.255098 -0.954616	C -3.752426 0.503842 1.251385
C 2.569528 1.841179 -0.908179	C -2.376162 0.484838 1.141621
F -0.767857 -0.286096 -0.306325	F 1.650623 -0.708404 -1.754461
F -3.107496 -1.524305 -0.462888	F 4.280043 -0.827387 -1.940699
C -5.526534 -0.220514 -0.042655	C 5.992887 0.293404 -0.045728

F -5.334954 2.489787 0.568767	F 4.732601 1.564301 2.103772
F -2.995850 3.748781 0.723424	F 2.083374 1.698934 2.323649
F 1.242369 0.265252 2.075613	F -2.067685 0.427852 -2.469485
F 3.723655 -0.754603 2.038709	F -4.717865 0.492637 -2.230474
C 5.549443 -0.182865 0.003359	C -5.995019 0.541267 0.257548
F 4.619437 1.570747 -1.960314	F -4.298037 0.512488 2.471233
F 2.187377 2.687289 -1.863415	F -1.667803 0.452041 2.268993
C 6.661955 -0.665119 -0.028158	C -7.196611 0.566086 0.367479
I 8.480438 -1.455920 -0.080934	I -9.188094 0.607195 0.549538
C -6.590680 -0.794369 -0.116810	C 7.193911 0.227713 -0.144489
I -8.329257 -1.731121 -0.237830	I 9.184459 0.118922 -0.307989

**E2-A3** M06L-D3/def2-TZVP	B3LYP-D3//def2-TZVP
28	28
Energy = -15357.2435438	Energy = -15358.0519145
C 1.642518 1.520426 0.116804	C -0.275389 -1.743595 -0.006557
N 0.452536 2.039450 0.167407	N -0.508599 -0.369584 -0.009310
N -0.640037 2.578828 0.289987	N 0.508599 0.369584 -0.009310
C -1.802731 1.779359 0.220616	C 0.275389 1.743595 -0.006557
C -1.872086 0.413217 -0.085438	C -0.959222 2.423685 0.027067
C -3.084926 -0.231147 -0.171446	C -1.009292 3.803365 0.030229
C -4.294724 0.443924 0.044243	C 0.147202 4.593171 -0.000481
C -4.219670 1.807803 0.349739	C 1.372072 3.918987 -0.033506
C -3.009064 2.459427 0.431274	C 1.434465 2.540857 -0.035753
C 2.083184 0.587742 1.092708	C 0.959222 -2.423685 0.027067
C 3.355931 0.081608 1.070917	C 1.009292 -3.803365 0.030229
C 4.264261 0.373162 0.039411	C -0.147202 -4.593171 -0.000481
C 3.806895 1.255098 -0.954616	C -1.372072 -3.918987 -0.033506
C 2.569528 1.841179 -0.908179	C -1.434465 -2.540857 -0.035753
F -0.767857 -0.286096 -0.306325	F -2.117223 1.767680 0.061108
F -3.107496 -1.524305 -0.462888	F -2.204273 4.400847 0.064187
C -5.526534 -0.220514 -0.042655	C 0.078450 6.002299 0.002163
F -5.334954 2.489787 0.568767	F 2.508890 4.619628 -0.063802
F -2.995850 3.748781 0.723424	F 2.635771 1.963990 -0.068361
F 1.242369 0.265252 2.075613	F 2.117223 -1.767680 0.061108
F 3.723655 -0.754603 2.038709	F 2.204273 -4.400847 0.064187

C	5.549443	-0.182865	0.003359	C	-0.078450	-6.002299	0.002163
F	4.619437	1.570747	-1.960314	F	-2.508890	-4.619628	-0.063802
F	2.187377	2.687289	-1.863415	F	-2.635771	-1.963990	-0.068361
C	6.661955	-0.665119	-0.028158	C	-0.019421	-7.207752	0.004372
I	8.480438	-1.455920	-0.080934	I	0.078225	-9.205484	0.008066
C	-6.590680	-0.794369	-0.116810	C	0.019421	7.207752	0.004372
I	-8.329257	-1.731121	-0.237830	I	-0.078225	9.205484	0.008066

V. Crystallographic details

Single-crystals were mounted using a microfabricated polymer film crystal-mounting tool (dual-thickness MicroMount, MiTeGen) using low viscosity oil (perfluoropolyalkylether; viscosity 1800 cSt, ABCR) to reduce the X-ray absorption and scattering. A Bruker D8 Venture single-crystal X-ray diffractometer with area detector using Cu K_α (λ = 1.54178 Å) radiation was used for data collection at the temperature stated for each compound. Multiscan absorption corrections implemented in SADABS[12] were applied to the data. The structures were solved by intrinsic phasing (SHELXT-2013)[13] and refined by full-matrix least-squares methods on F^2 (SHELXL-2014).[14] The hydrogen atoms were placed at calculated positions and refined by using a riding model. CCDC 1936418 (**U1···A2**) contains the supplementary crystallographic data for this paper. These data can be obtained free of charge from The Cambridge Crystallographic Data Centre.

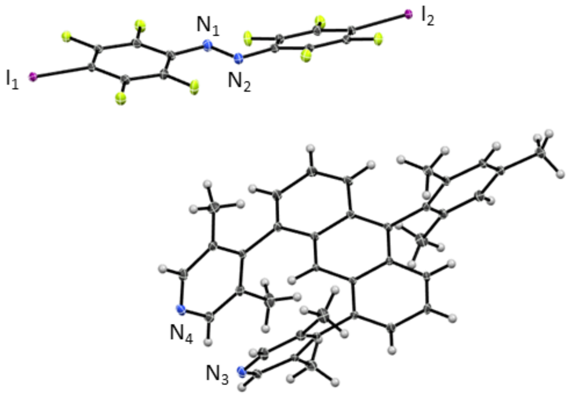

Figure S15. Single-crystal X-ray data of **U1···A2** showing the asymmetric unit with thermal ellipsoids set at 50% probability. The structure was measured at 120 K and solved in the monoclinic space group $P2_1/n$ with R_{Int} = 0.0398, R_1 = 0.0242 and wR_2 = 0.0675. Selected bond lengths [Å]: I1–N3 2.7810(2), I2–N4 2.816(2), N1–N2 1.148(3).

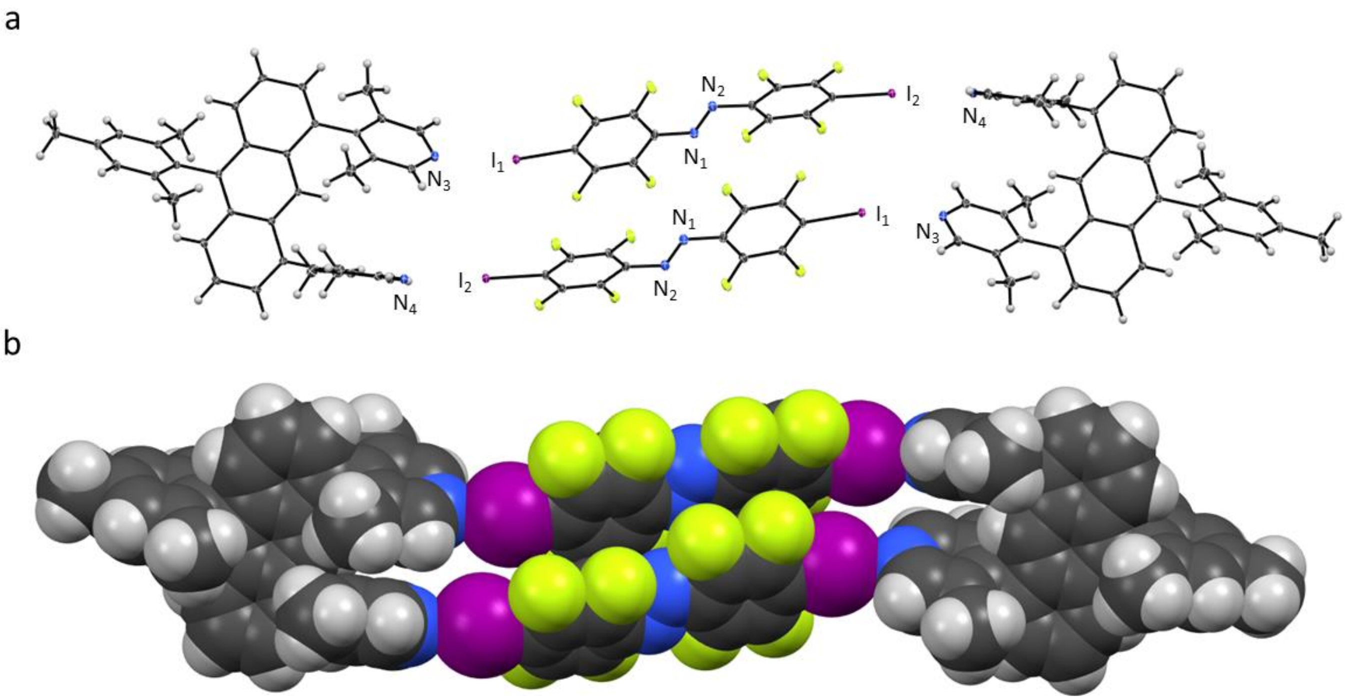

Figure S16. Single-crystal X-ray data of **U1···A2**, a) with thermal ellipsoids set at 50% probability and b) showing a space-filling model.

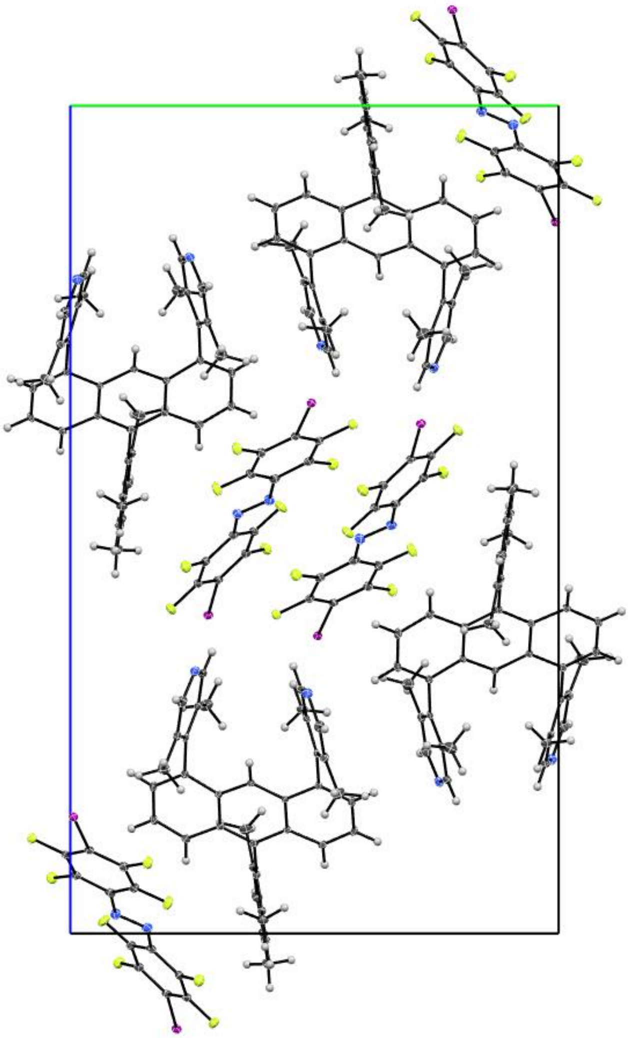

Figure S17. View of the unit cell of **U1···A2** along the crystallographic *a* axis. The tetrafluorinated azobenzenes **A2** engage in very distant lamellar 2D π-stacking with each other, whereas anthracene building blocks **U1** engage in various CH···π interactions CH···π interactions in the range from 2.87–3.51 Å by a self-complementing head-to-tail arrangement. The fluorinated and the non-fluorinated part are separated and engage in various C-H···F contacts such as F12···H33 2.86, F11···H45 2.69, F5···H41B 2.60, F5···H38 2.80, F5···H45 2.72, F2···H33C 2.86 and F3···H20 3.07 Å, also originating from the protruding methyl groups.

V. References

[1] Bléger, D.; Schwarz, J.; Brouwer, A. M.; Hecht, S. *J. Am. Chem. Soc.* **2012**, *134*, 20597–20600.

[2] Bushuyev, O. S.; Tomberg, A.; Friščić, T.; Barrett, C. *J. Am. Chem. Soc.* **2013**, *135*, 12556–12559.

[3] Nieland, E.; Topornicki, T; Kunde, T; Schmidt, B. M. *Chem. Commun.* **2019**, 55, 8768–8771.

[4] Benedetta, M.; *WIREs Comput. Mol. Sci.* **2012**, *2*, 386–404.

[5] Frisch, M. J. *et al.*; Gaussian16 Revision B.01. (2016).

[6] Knie, C.; Utecht, M.; Zhao, F.; Kulla, H.; Kovalenko, S.; Brouwer, A. M.; Saalfrank, P.; Hecht, S.; Bléger, D.; *Chem. Eur. J.* **2014**, *20*, 16492–16501.

[7] Rietze, C.; Titov, E.; Lindner, S.; Saalfrank, P.; *J. Phys. Condens. Matter* **2017**, *29*, 314002.

[8] Liu, X.-M.; Jin, X.-Y.; Zhang, Z.-X.; Wang, J.; Bai, F.-Q.; *RSC Adv.* **2018**, *8*, 11580–11588.

[9] Canneaux, S.; Bohr, F.; & Henon, E.; *J. Comput. Chem.* **2014**, *35*, 82–93.

[10] Piccini, G.; Alessio, M.; Sauer, J.; *Angew. Chem. Int. Ed. Engl.* **2016**, *55*, 5235–5237.

[11] Hübschle, C. B.; Dittrich, B.; *J. Appl. Cryst.* **2011**, *44*, 238–240.

[12] Sheldrick, G. M.; SADABS, program for empirical absorption correction of area detector data, University of Göttingen, Göttingen, 1996.

[13] Sheldrick, G. M.; SHELXT-2013, Program for Crystal Structure Solution, University of Göttingen, Göttingen, 2013.

[14] Sheldrick, G. M.; SHELXL-2014, Program for Crystal Structure Refinement, University of Göttingen, Göttingen, 2014.
